# TNF-Overexpression in Borna Disease Virus-Infected Mouse Brains Triggers Inflammatory Reaction and Epileptic Seizures

**DOI:** 10.1371/journal.pone.0041476

**Published:** 2012-07-25

**Authors:** Katharina Kramer, Dirk Schaudien, Ulrich L. M. Eisel, Sibylle Herzog, Jürgen A. Richt, Wolfgang Baumgärtner, Christiane Herden

**Affiliations:** 1 Department of Pathology, University of Veterinary Medicine, Hannover, Germany; 2 Department of Molecular Neurobiology, University of Groningen, Groningen, The Netherlands; 3 Institute of Virology, Justus-Liebig-University, Gießen, Germany; 4 Department of Diagnostic Medicine/Pathobiology, Kansas State University, Manhattan, Kansas, United States of America; Auburn University, United States of America

## Abstract

Proinflammatory state of the brain increases the risk for seizure development. Neonatal Borna disease virus (BDV)-infection of mice with neuronal overexpression of tumor necrosis factor-α (TNF) was used to investigate the complex relationship between enhanced cytokine levels, neurotropic virus infection and reaction pattern of brain cells focusing on its role for seizure induction. Viral antigen and glial markers were visualized by immunohistochemistry. Different levels of TNF in the CNS were provided by the use of heterozygous and homozygous TNF overexpressing mice. Transgenic TNF, total TNF (native and transgenic), TNF-receptor (TNFR1, TNFR2), IL-1 and N-methyl-D-aspartate (NMDA)-receptor subunit 2B (NR2B) mRNA values were measured by real time RT-PCR. BDV-infection of TNF-transgenic mice resulted in non-purulent meningoencephalitis accompanied by epileptic seizures with a higher frequency in homozygous animals. This correlated with lower weight gain, stronger degree and progression of encephalitis and early, strong microglia activation in the TNF-transgenic mice, most obviously in homozygous animals. Activation of astroglia could be more intense and associated with an unusual hypertrophy in the transgenic mice. BDV-antigen distribution and infectivity in the CNS was comparable in TNF-transgenic and wild-type animals. Transgenic TNF mRNA-expression was restricted to forebrain regions as the transgene construct comprised the promoter of NMDA-receptor subunit2B and induced up-regulation of native TNF mRNA. Total TNF mRNA levels did not increase significantly after BDV-infection in the brain of transgenic mice but TNFR1, TNFR2 and IL-1 mRNA values, mainly in the TNF overexpressing brain areas. NR2B mRNA levels were not influenced by transgene expression or BDV-infection. Neuronal TNF-overexpression combined with BDV-infection leads to cytokine up-regulation, CNS inflammation and glial cell activation and confirmed the presensitizing effect of elevated cytokine levels for the development of spontaneous epileptic seizures when exposed to additional infectious noxi.

## Introduction

Epileptic seizures might arise after various brain insults either already in infancy or later in life ranging from trauma, stroke, brain tumors up to infections with or without febrile seizures and status epilepticus. All of these events can trigger inflammatory processes in the brain and this proinflammatory state represents a risk factor for seizure induction and maintenance [1]–[6]. There is also increasing evidence that beside central nervous system (CNS) inflammation with enhanced cytokine levels, activation and dysfunction of microglia and astrocytes cells with cytokine secretion play an important role for occurrence of seizures in several different epilepsy forms [1]–[10]. Most reports concentrate on the potential role of TNF, IL-1 or Interleukin 6 (IL-6) .in the epileptogenic process [1]–[6], [10]. Functional disturbances of the immune system such as autoimmune CNS-diseases can also be associated with epileptic seizures and seizures themselves are able to stimulate synthesis of proinflammatory and proconvulsive cytokines [4], [8], [10]–[12]. Cytokines are also able to interfere with excitability of neurons rapidly by interaction with glutamate and GABA receptors, extracellular glutamate levels and blood brain barrier and on a more prolonged base by effects on gene transcription [2], [6], [13]. However, despite substantial progress in epilepsy research, in more than 66% of human epileptic patients the cause for seizures remains unknown. Virus infections of the CNS, e.g. herpesviruses, can serve as environmental trigger because about 50% of humans with encephalitis develop seizures and have a higher risk to develop epilepsy later after recovery [14]–[19]. Recent experimental infections with picornavirus, herpes simplex virus, West Nile virus, measles virus or lymphocytic choriomeningitis virus (LCMV) have substantiated this assumption [14], [15], [20]–[23].

Natural and experimental infection with Borna disease virus (BDV), a neurotropic, single stranded, negative sense RNA virus, typically causes a severe neurological disorder (Borna disease, BD) due to a progressive non-purulent meningoencephalitis accompanied by virus persistence in the CNS [24]–[26]. Seizures might occur in later stages of the natural disease. Main natural hosts are horses and sheep, but in experimental settings, many warm-blooded animal species can be easily infected. Recently, new avian bornaviruses have been detected in psittacine birds suffering from proventricular dilatation disease [27], [28]. The association of BDV-infection with human psychiatric diseases has been discussed controversially; only the presence of BDV serum antibodies in such patients is widely accepted to date [25], [26], [29], [30].

Experimental BDV-infection of rodents is of great value to study the virally induced immune-mediated neuropathologic processes and the response of brain cells to inflammation and virus infection [24]–[26], [31]. Only neonatal mice develop clinical disease after BDV-infection but clinical signs differ notably between various mouse strains. Typical clinical signs in mice are hunched posture, rough fur, weight loss and tilted head. Importantly, seizures have yet not been noted in BDV-infected mice. In BDV-infection of mice and rats, the invasion of immune cells into the CNS is regularly accompanied by glial cell activation, up-regulation of proinflammatory cytokines and CD4+ and CD8+ T-cells play a crucial role in this process [25], [31]–[34]. However, clearance of BDV or cytolysis is generally not observed after experimental infection of rodents and viral spread within the mouse brain is not influenced by cytokines (e.g. interferon γ [IFNγ]) [25], [34]–[36]. There is evidence that especially TNF represents a critical cytokine for the antiviral immune response in various neurotropic virus infections, e.g. rabies virus, West Nile virus and respiratory syncytial virus [37]–[41]. Interestingly, TNF can also act as proconvulsive or anticonvulsive factor depending on signalling via TNF receptor 1 or 2 and contribute to a reduced seizure threshold in models mimicking viral and bacterial infections in children with respective long-term changes [2], [4], [6], [10], [13], [20], [42]–[45]. TNF plays already a role in physiological brain homeostasis since brains cells can secrete TNF and express TNF-receptors [3], [4], [46]–[48].

Due to the versatile roles of TNF, several transgenic mice expressing TNF under CNS-specific and non CNS-specific promoters have been established so far [49]–[51]. The transgenic mouse line used in the present study expressed the murine TNF under the control of the promoter of the N-methyl-D-aspartate (NMDA) receptor subunit NR2B/ε2 and, therefore, demonstrated a moderate TNF-overexpression restricted to forebrain regions of the adult CNS [52]. These mice do not show clinical signs or brain lesions despite a mild activation of microglia. Importantly, TNF-overexpression is selectively active in defined brain regions where BDV-replication takes place thereby affecting the BDV-induced neuropathology. Homozygous and heterozygous transgenic mice expressing different TNF-levels were infected with BDV and the effects of TNF on clinical signs, inflammatory response, cytokine induction, reaction pattern of brain cells and viral spread in the CNS were analyzed. We describe a new model that allows studying the complex interaction between a multifunctional cytokine such as TNF, brain inflammation, reaction of brain cells and neurotropic virus infection. This will improve our understanding of the presensitizing effects of elevated cytokine levels in the CNS and the reciprocal neuroimmune interactions during brain infections focusing on their impact for seizure induction.

## Results

### 1. Clinical Examination

TNF-transgenic mice, especially homozygous mice, showed a lower weight gain and developed spontaneous epileptic seizures.

**Figure 1:**
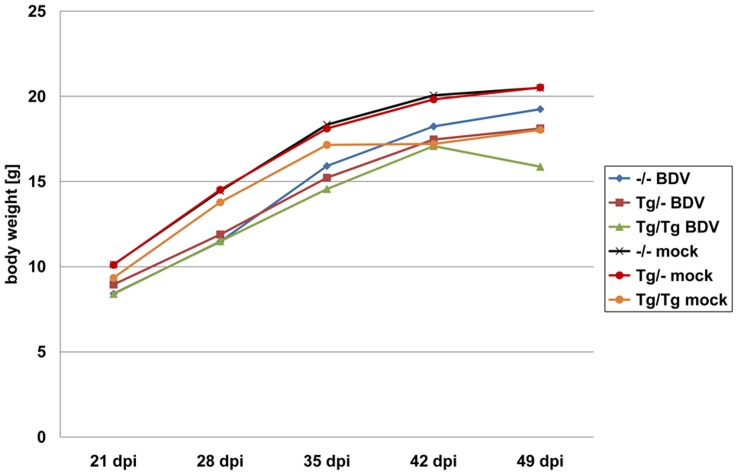
Development of weight gain in mock-infected and BDV-infected mice. TNF-transgenic animals gained less weight than the wild-type mice after BDV-infection. In general, all BDV-infected mice groups exhibited al lower weight gain when compared to mock-infected animals. dpi: days post infection, –/–: non-transgenic mice, Tg/–: heterozygous transgenic mice, Tg/Tg: homozygous transgenic mice, BDV: BDV-infected, mock: mock-infected, arithmetic mean.

All BDV- and mock-infected non-transgenic and TNF-transgenic mice gained **weight** between 21 to 42 dpi, but the amount of weight gain differed significantly between the groups (Fig. 1). In general, a lower weight gain was found in homozygous TNF-transgenic mice compared to heterozygous and non-transgenic counterparts after BDV-infection (p = 0.0128, p = 0.0450, respectively, table S1). In addition, homozygous BDV-infected mice put on less weight than respective mock-infected mice (p = 0.0275, table S1). Notably, a significantly lower weight gain was also found in BDV-infected non-transgenic mice compared to mock-infected animals (p = 0.0101, table S1). Significant influence of the status of infection (p = 0.0091, table S1) on the mean weight gain (p = 0.0217) were found in the global statistical evaluation using the repeated measurement analysis of variances including the parameter transgenic status and status of infection. In addition, the transgenic status showed only a mild influence (p = 0.0814, table S1).

The clinical-neurological tests (activity, gait on the grid and on a flat surface, ability to balance on a stick, motoric activity, sensitivity, grasp and withdrawal reflex) revealed only mild differences without statistical significance between homo- and heterozygous TNF-transgenic and non-transgenic BDV-infected mice and the respective mock-infected or non-infected cohort (tables S2).

**Figure 2:**
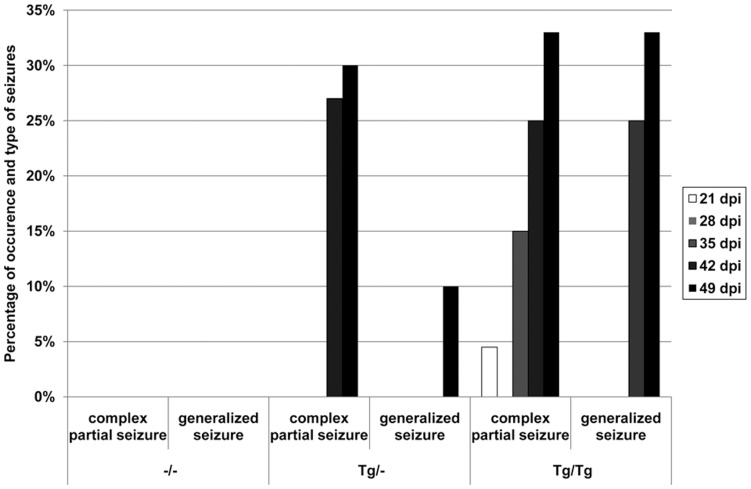
Frequency of seizures in BDV-infected mice. Spontaneous epileptic seizures were exclusively observed in BDV-infected transgenic animals and appeared more frequently in homozygous transgenic BDV-infected mice starting 21 dpi. Seizures were first noted 42 dpi in heterozygous transgenic animals. dpi: days post infection, –/–: non-transgenic mice, Tg/–: heterozygous transgenic mice, Tg/Tg: homozygous transgenic mice

Interestingly, **spontaneous epileptic seizures** were exclusively observed in BDV-infected transgenic animals during clinical examinations. These seizures could be differentiated in complex partial and generalized seizures. The complex partial and generalized seizure lasted up to 30 and 50 sec, respectively. The complex partial seizures started with a tonic phase, in which the animals head turned rigidly upwards and back simultaneously with bilateral forelimb extension. The longer lasting generalized seizures began similarly, but the 10–20 sec lasting tonic phase was followed by a tonic-clonic phase. This phase was characterized by a rapid bilateral hind limb thrusting leading to vigorous bouncing. 100% of the mice suffering from generalized seizures died during the tonic-clonic seizures. Seizures appeared more frequently in homozygous transgenic BDV-infected mice starting at 21 dpi. In contrast, seizures were first seen at 42 dpi in heterozygous transgenic animals (Fig. 2). No form of seizures was observed in non-transgenic BDV-infected mice or any non-infected TNF transgenic or mock-infected TNF transgenic animal.

All three BDV-infected mouse groups showed mild gait abnormalities and problems balancing on a stick at 35 dpi. All mock-infected mice, independent of their transgenic status, displayed no clinical signs and were ranged into normal scores of the clinical-neurological tests.

### 2. Expression of Transgenic TNF (TNFtg) mRNA, Total TNF (TNFto) mRNA, IL-1 mRNA, TNFR1 mRNA, TNFR2 mRNA and NR2B mRNA

TNFtg mRNA was present only in the transgenic animals. Total TNF (TNFto) mRNA values were significantly higher in transgenic mice with highest copy numbers in homozygous mice indicating that TNFtg induced additional native TNF mRNA expression. No significant increase of any TNF mRNA was detected in transgenic animals after BDV-infection. IL-1 mRNA levels increased significantly in all animal cohorts after BDV-infection. After BDV-infection IL-1, TNFR1 and TNFR2 mRNA levels increased significantly predominantly in brain areas with TNF-overexpression. Neither transgene expression nor BDV-infection caused significant changes in NR2B mRNA expression.

All mRNA values were analyzed 42 dpi in highly TNF-overexpressing areas (cortex cerebri, striatum, hippocampus), compared to a non-expressing area (cerebellum) and subjected to further statistical analysis (tables S3, S4).

**Figure 3:**
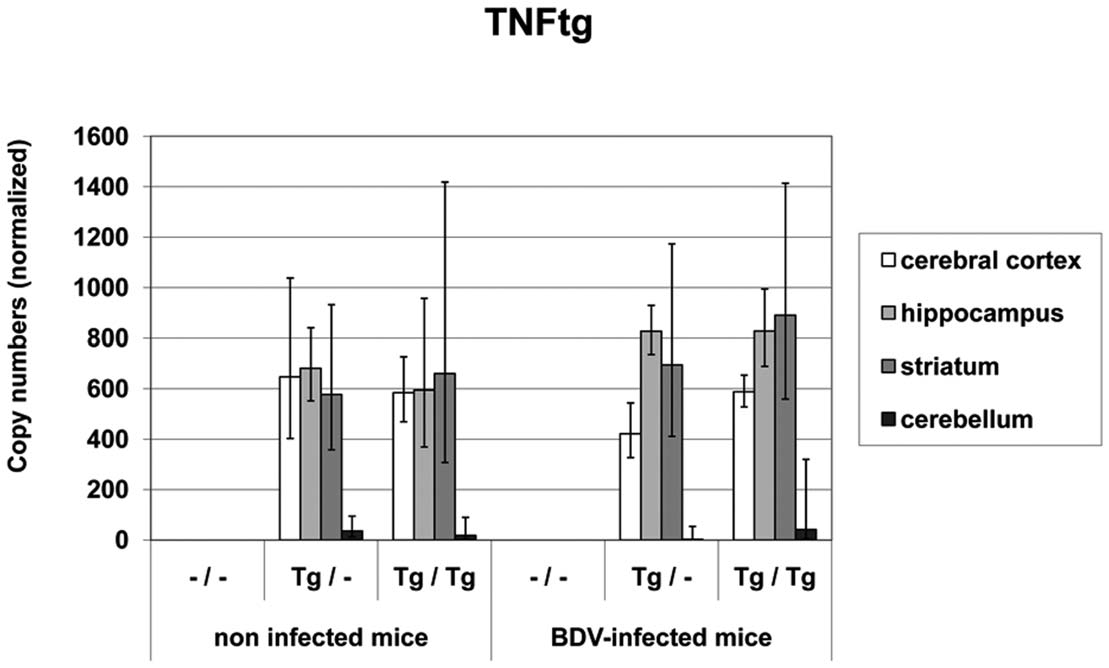
Transgenic TNF (TNFtg) mRNA values in different brain areas in TNF-transgenic and wild-type mice. TNFtg mRNA was present only in the transgenic animals. TNFtg mRNA copy numbers were significantly higher in the cerebral cortex, hippocampus and striatum when compared to the cerebellum in Tg/– and Tg/Tg animals regardless of BDV-infection. No TNFtg mRNA was detected in the wild-type mice in any brain area. dpi: days post infection, –/–: non-transgenic mice, Tg/–: heterozygous transgenic mice, Tg/Tg: homozygous transgenic mice, geometric mean, bar: distribution factor

**TNFtg mRNA** levels yielded in average between 5.77×10^2^ and 6.82×10^2^ normalized copies in the non-infected heterozygous mice and between 5.85×10^2^ and 6.61×10^2^ normalized copies in the non-infected homozygous animals. After BDV-infection, TNFtg mRNA copy numbers between 4.22×10^2^ and 8.27×10^2^ in the heterozygous mice and between 5.87×10^2^ and 8.9×10^2^ in the homozygous mice in the cerebral cortex, hippocampus and striatum were measured (Fig. 3). Normalized copy numbers in the cerebellum ranged from 2 to 42 in the transgenic mouse groups regardless of BDV-infection. Therefore, TNFtg mRNA copy numbers were significantly higher in the cerebral cortex, hippocampus and striatum when compared to the cerebellum in Tg/– non-infected and infected mice (p<0.01) and in Tg/Tg non-infected mice (p<0.01) and infected animals (p<0.05). No TNFtg mRNA was detected in the wild-type mice in any brain area.

**Figure 4:**
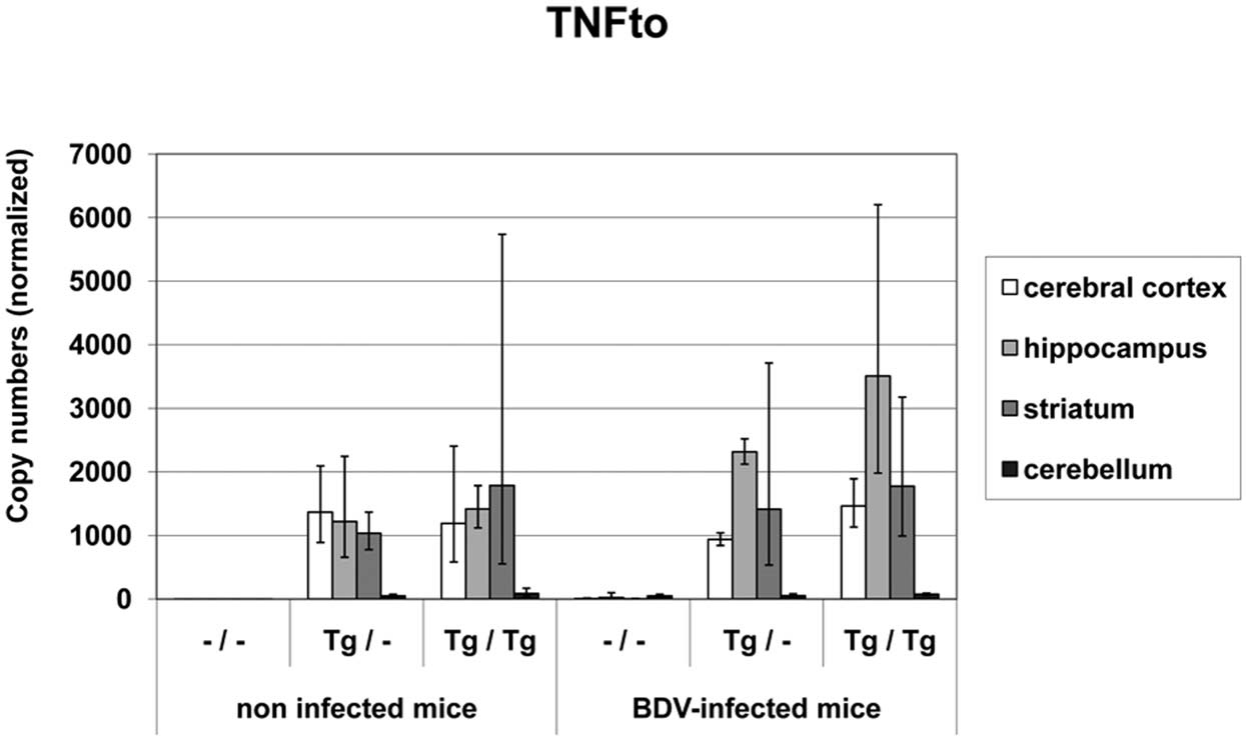
Total TNF (TNFto) mRNA values in different brain areas in TNF-transgenic and wild-type mice. TNFto mRNA values were significantly higher in transgenic mice and consisted of approximately 50% native TNF mRNA indicating that TNFtg induced native TNF mRNA expression. Highest copy numbers were found in homozygous mice. No significant increase of any TNF mRNA was detected in transgenic animals after BDV-infection but values were highest in the hippocampus. dpi: days post infection, –/–: non-transgenic mice, Tg/–: heterozygous transgenic mice, Tg/Tg: homozygous transgenic mice, geometric mean, bar: distribution factor.

**TNFto mRNA** copy numbers consisted of transgenic and native TNF mRNA. At day 42 p.i., non-infected wild-type mice did not express hardly any TNFto mRNA but values increased up to 49 copies after BDV-infection only in the cerebellum. In the heterozygous non-infected mice TNFto mRNA values ranged between 1.04×10^3^ and 1.37×10^3^ and between 1.19×10^3^ and 1.79×10^3^ copies in the homozygous non-infected mice in the TNF overexpressing areas (Fig. 4). Interestingly, after BDV-infection, TNFto mRNA levels reached at maximum 2.31×10^3^ copies in the Tg/– animals and 3.09×10^3^ in the Tg/Tg mice in the hippocampus with lower levels in the other transgene expressing areas (Tg/–: 9.4×10^2^−1.41×10^3^ copy numbers, Tg/Tg: 1.46×10^3^−1.77×10^3^ copy numbers; Fig. 4). Normalized copy numbers in the cerebellum ranged from 53 to 99 in the transgenic mouse groups regardless of BDV-infection. Similar as TNFtg mRNA expression, total TNF mRNA copy numbers were significantly higher in the cerebral cortex, hippocampus and striatum when compared to the cerebellum in Tg/– and Tg/Tg non-infected and infected mice (p<0.0001). By statistical analysis a significant increase of TNFto mRNA could not be documented in any brain area in both transgenic cohorts after BDV-infection when compared to the respective non-infected cohort. This was only found in the wild-type mice (p<0.0001). However, TNFto mRNA levels were significantly higher in transgenic mice than in the wild-type animals and even significantly higher in the homozygous mice compared to the heterozygous cohort in a global comparison. Moreover, transgenic TNF was able to induce native TNF mRNA.

**Figure 5:**
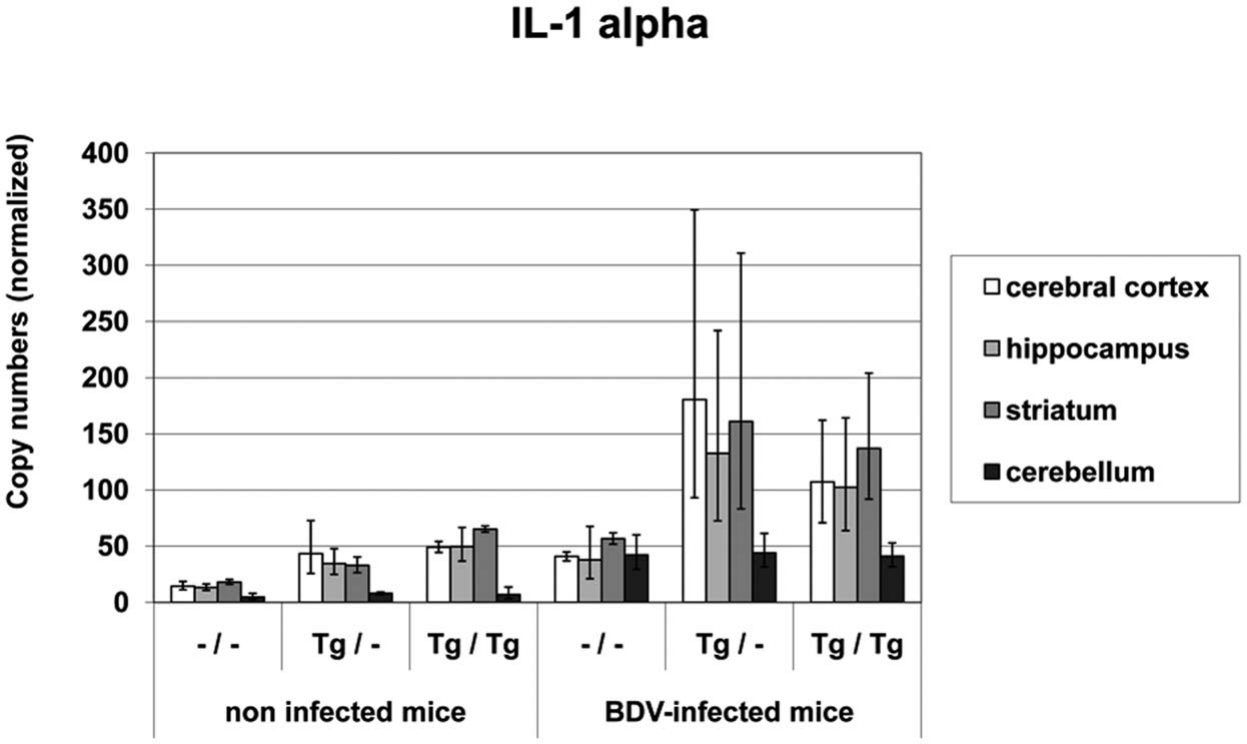
IL-1 mRNA values in different brain areas in TNF-transgenic and wild-type mice. IL-1 mRNA levels increased significantly in all animal cohorts after BDV-infection but copy numbers were significantly higher in the transgenic mice when compared to wild-type mice regardless of infection status. Highest values were detected TNF overexpressing areas. dpi: days post infection, –/–: non-transgenic mice, Tg/–: heterozygous transgenic mice, Tg/Tg: homozygous transgenic mice, geometric mean, bar: distribution factor.

In all mouse groups, a low constitutive **IL-1 mRNA** expression was detected with values between 5 and 50 copy numbers. After BDV-infection, IL-1 mRNA levels increased significantly in all animal cohorts when compared to the respective non-infected mouse group (copy numbers in the different brain areas:–/–: 38 – 57, p<0.005; Tg/–: 1.33×10^2^−1.8×10^2^, p<0.001; Tg/Tg: 1.07×10^2^−1.37×10^2^; p<0.05; Fig. 5). Copy numbers were significantly higher in the Tg/– and Tg/Tg mice when compared to wild-type mice regardless of infection status in the cerebral cortex, hippocampus and striatum (p<0.05−p<0.001). In the transgenic non-infected and infected mice significantly lowest copy numbers of IL-1 mRNA were measured in the cerebellum (p<0.05−p<0.001).

**Figure 6:**
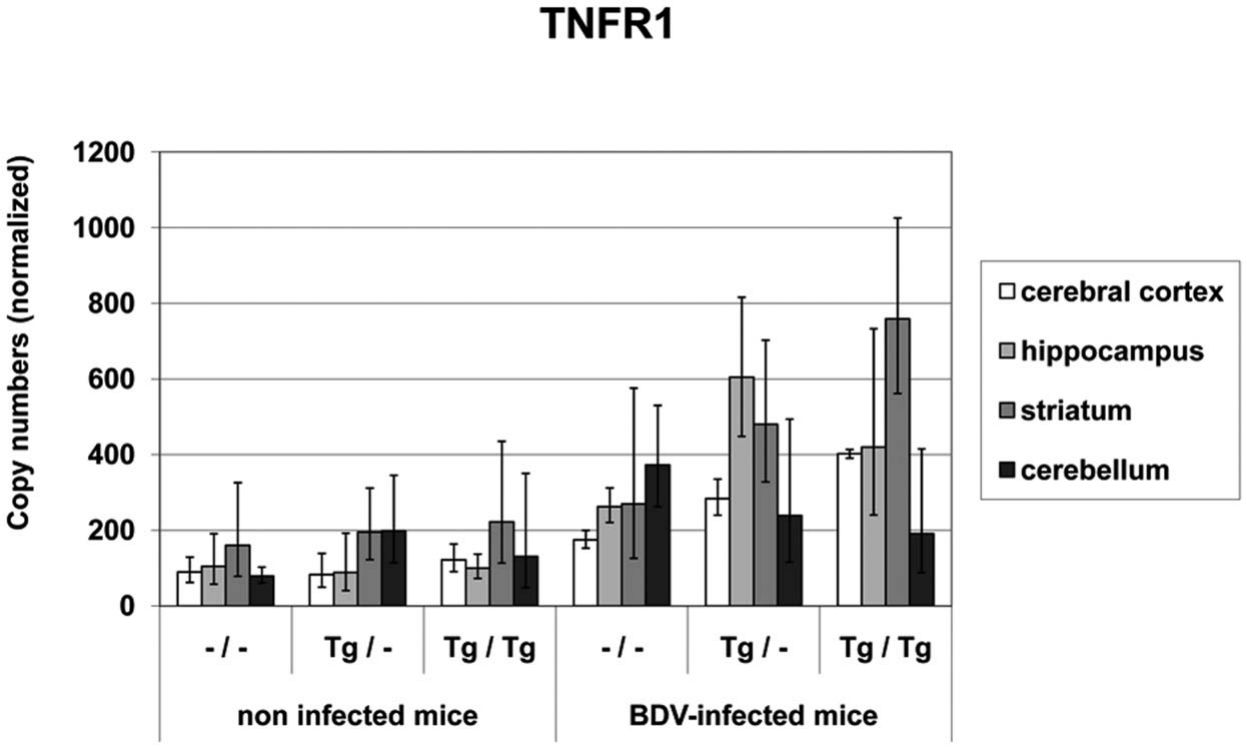
TNFR1 mRNA values in different brain areas in TNF-transgenic and wild-type mice. After BDV-infection TNFR1 mRNA levels increased significantly in transgenic mice, mainly in the brain areas with TNF-overexpression. dpi: days post infection, –/–: non-transgenic mice, Tg/–: heterozygous transgenic mice, Tg/Tg: homozygous transgenic mice, geometric mean, bar: distribution factor.

Constitutive **TNFR1 mRNA** expression was found in all non-infected mouse groups with values ranging between 79 and 2.22×10^2^ copies. After BDV-infection, TNFR1 mRNA levels increased significantly in the hippocampus (p = 0.0391) and in the cerebellum (p = 0.0009) in the wild-type mice and in the transgenic areas cerebral cortex, hippocampus and striatum in the Tg/– and Tg/Tg mice (p = 0.0431–p<0.0001, Fig. 6). This indicated that TNFR1 mRNA copies were highest in areas with high amounts of TNFtg and TNFto mRNA.

**Figure 7:**
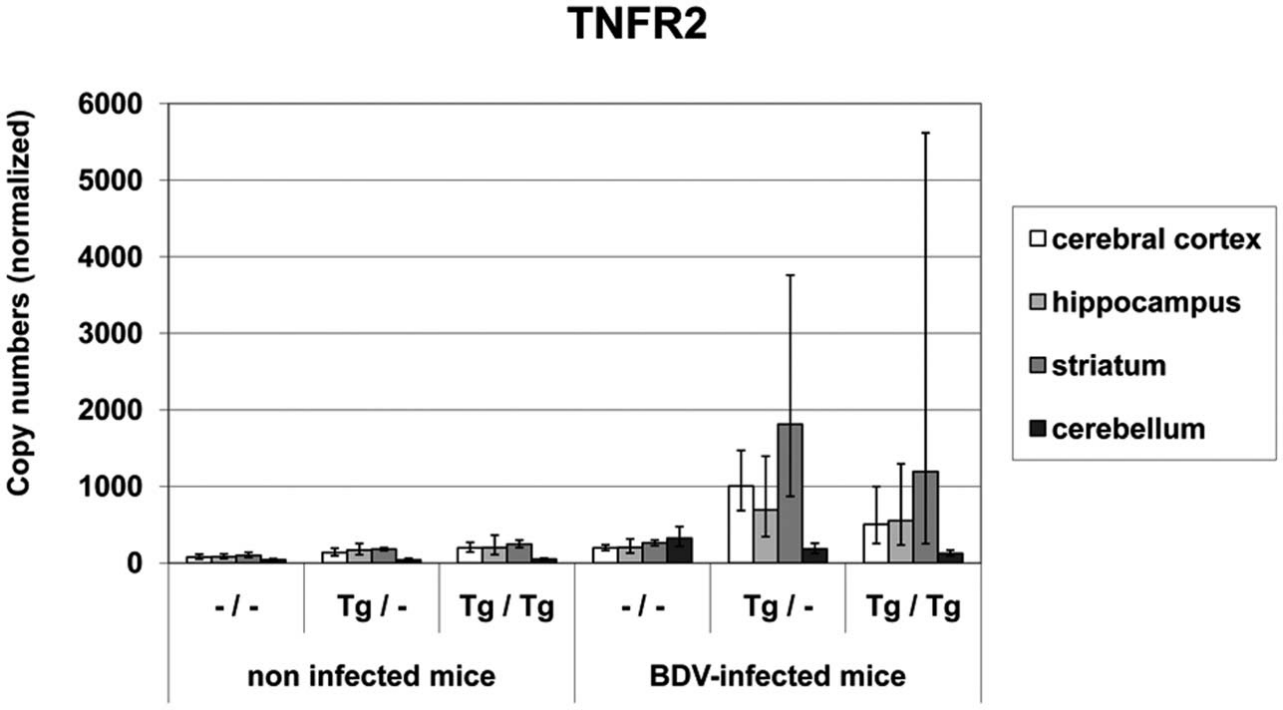
TNFR2 mRNA values in different brain areas in TNF-transgenic and wild-type mice. After BDV-infection TNFR2 mRNA levels increased significantly in the transgenic mice, mainly in the brain areas with TNF-overexpression. dpi: days post infection, –/–: non-transgenic mice, Tg/–: heterozygous transgenic mice, Tg/Tg: homozygous transgenic mice, geometric mean, bar: distribution factor.

Constitutive **TNFR2 mRNA** values ranged between 44 and 2.47×10^2^ copies in the non-infected cohorts. After BDV-infection, a significant increase of TNFR2 mRNA levels was measured in all animal cohorts except for the hippocampus in the wild-type mice (copy numbers for the different brain areas: –/–: 1.44×10^2^−3.24×10^2^, Tg/–: 1.83×10^2^−1.81×10^3^, Tg/Tg: 1.26×10^2^−1.19×10^3^ copy numbers; p<0.05−p<0.001, Fig. 7). Non-infected and infected transgenic animals depicted significantly higher TNFR2 mRNA values in the TNF-overexpressing areas when compared to the cerebellum (p<0.01−p<0.0001).

**Figure 8:**
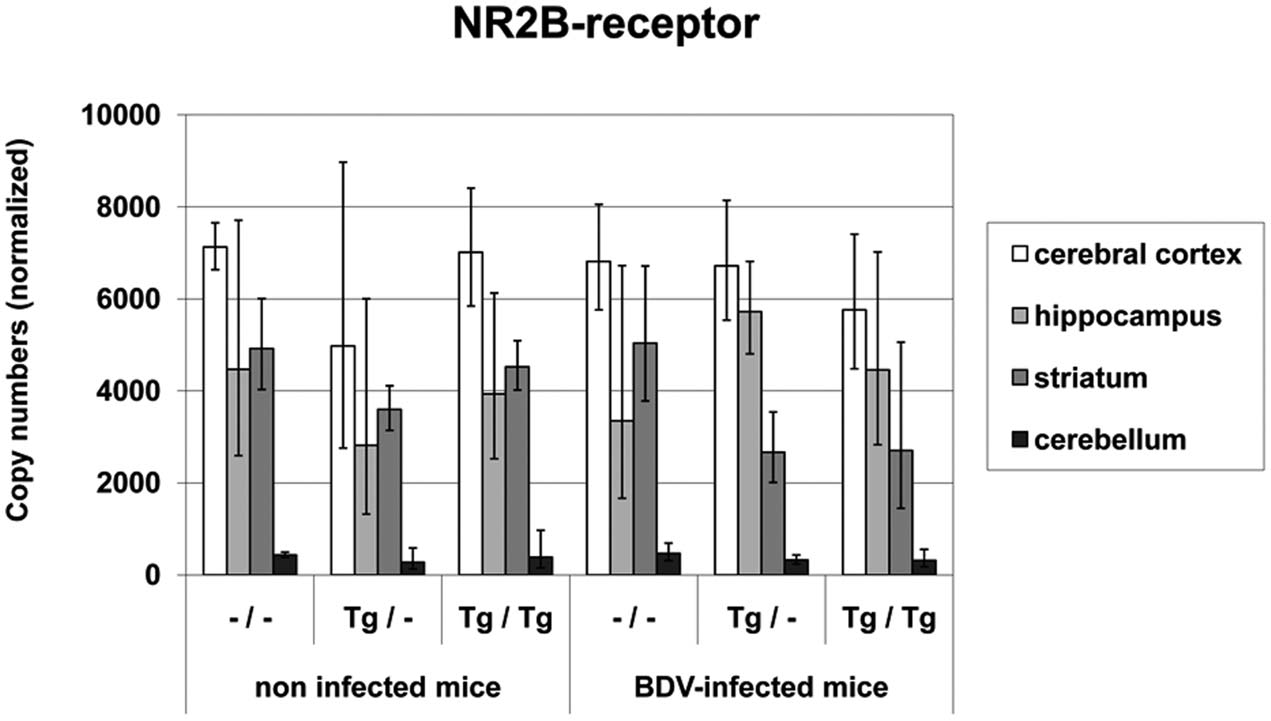
NR2B mRNA values in different brain areas in TNF-transgenic and wild-type mice. Neither transgene expression nor BDV-infection caused significant changes in NR2B mRNA expression. dpi: days post infection, –/–: non-transgenic mice, Tg/–: heterozygous transgenic mice, Tg/Tg: homozygous transgenic mice, geometric mean, bar: distribution factor.

Copy numbers of **NR2B mRNA** yielded between 3.9×10^2^ and 7.13×10^3^ in the non-infected cohorts and between 3.18×10^2^ and 6.81×10^3^ in the BDV-infected mouse groups. Significantly lowest copy numbers were found in the cerebellum regardless of transgene or infection status (p<0.0001, Fig. 8). Neither transgene expression nor BDV-infection caused significant changes in NR2B mRNA expression.

### 3. Inflammatory Reaction and Viral Infection of the Brain

The inflammatory reaction, microglia activation and type of astroglial reaction correlated strongly with the transgene status of the animals despite comparable viral spread and infectivity in transgenic and non-transgenic mice. Highest inflammatory scores and earliest, strongest microglia activation were found in the homozygous mice.

**Figure 9:**
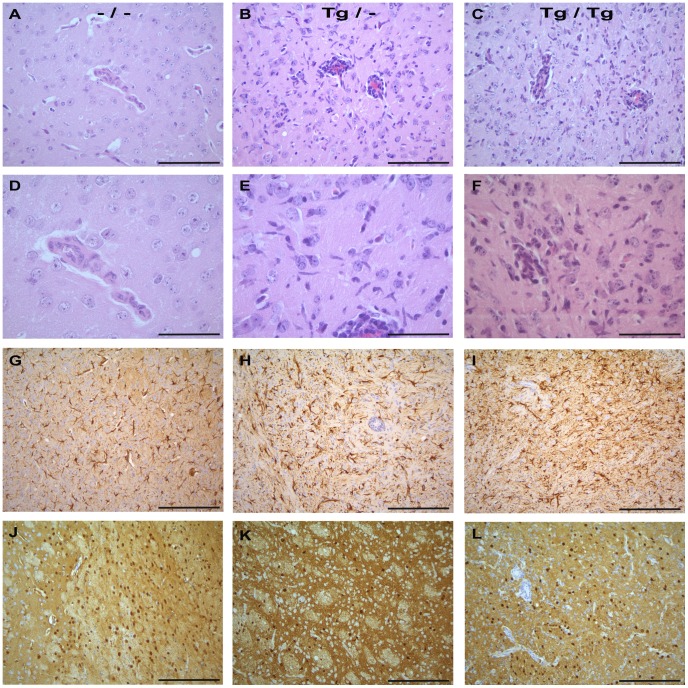
Inflammation, glial activation and viral distribution in the brain of BDV-infected TNF-transgenic and wild-type mice. A–F Hematoxylin and eosin staining: Mild inflammatory reaction and no microglia activation in non-transgenic BDV-infected mice (A, higher magnification in D). In contrast, moderate to severe inflammatory reaction and microglia activation in heterozygous (B, higher magnification in E) and homozygous (C, higher magnification in F) TNF-transgenic mice. TNF-overexpressing striatum, 42 dpi Hematoxylin and eosin staining was used to assess the degree of encephalitis and number of reactive microglia cells. **G–I GFAP-immunostaining:** Astrogliosis in all BDV-infected mice groups with more GFAP-positive astrocytes in the transgenic animals. GFAP-immunostaining was carried out to assess the number and morphology of activated astrocytes as GFAP expressing cells. TNF-overexpressing striatum, 42 dpi –/– **J–L Immunostaining for the viral nucleoprotein: **Comparable viral distribution within the brain in all BDV-infected mice groups. Immunostaining for the viral nucleoprotein was used to assess the number of BDV-infected cells. TNF-overexpressing striatum, 42 dpi –/–: non-transgenic mice, Tg/–: heterozygous transgenic mice, Tg/Tg: homozygous transgenic mice,scale bars: 100 µm (A–C; G–L); 50 µm (D–F).

**Figure 10:**
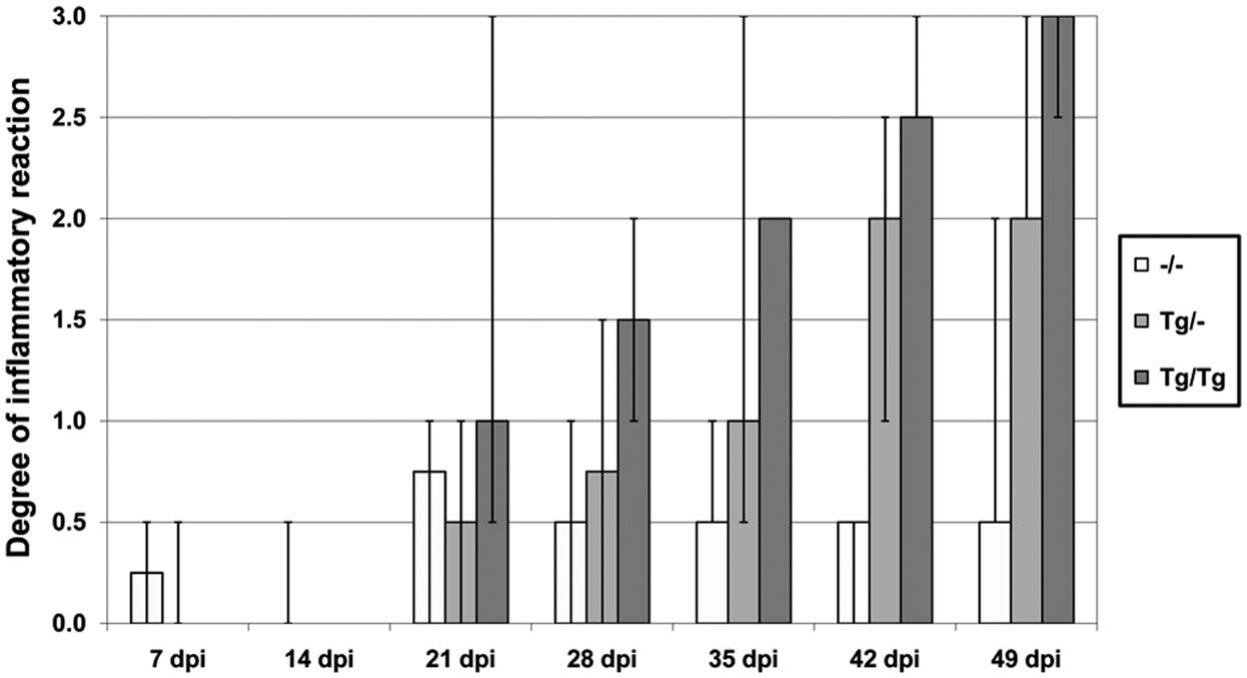
Degree of inflammatory reaction in the brain of BDV-infected animals. Wild-type mice showed a mild immune cell infiltration without progression during the investigation period whereas in both TNF-overexpressing transgenic mice groups a progressive severe non-purulent meningoencephalitis was noted. Highest inflammatory scores were found in the homozygous mice. Score for the inflammatory reaction: 0: no, 1: mild, 2: moderate, 3: severe inflammatory reaction; score for the astrogliosis: 0: no, 1: mild, 2: moderate, 3: severe astrogliosis, p.i.: post infection, –/–: non-transgenic mice, Tg/–: heterozygous transgenic mice, Tg/Tg: homozygous transgenic mice, median, bar: minimal/maximal value.

**Figure 11:**
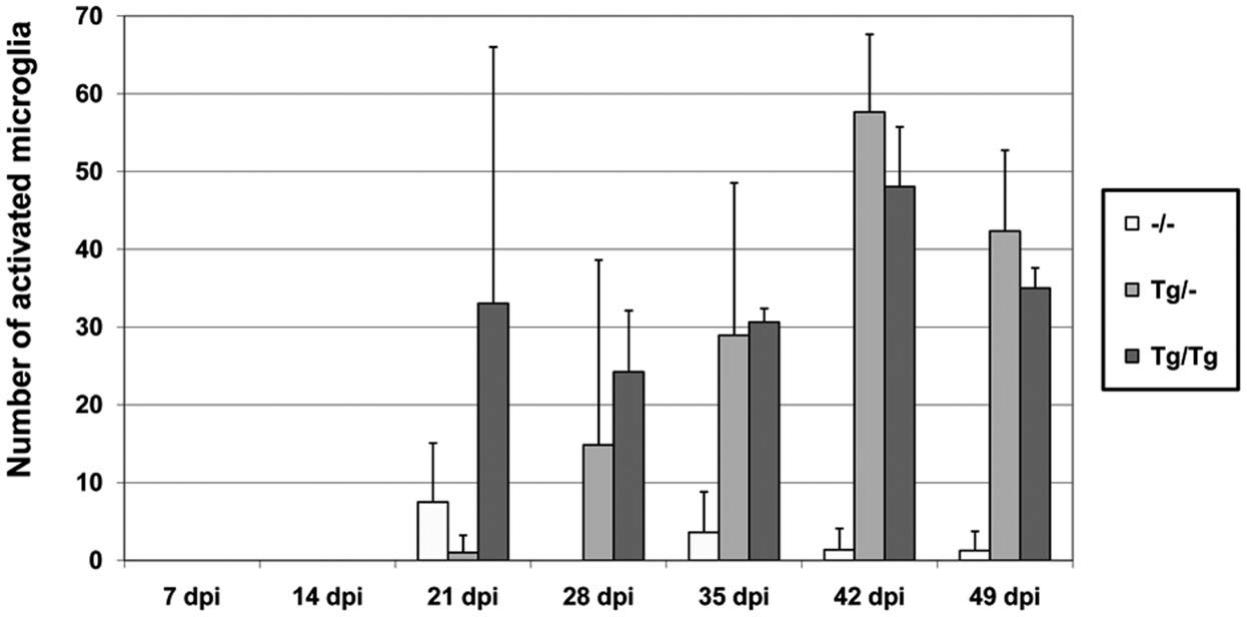
Average number of activated microglia in BDV-infected mice. Microglia increased significantly only in the TNF-transgenic BDV-infected mice starting 21 dpi. Earliest strongest microglia was found in the homozygous mice. dpi: days post infection, –/–: non-transgenic mice, Tg/–: heterozygous transgenic mice, Tg/Tg: homozygous transgenic mice, arithmetic mean, bar: minimal/maximal value.

Non-transgenic mice showed a mild **immune cell infiltration** without progression during the investigation period (Figs. 9A, 10). In contrast, in both TNF-overexpressing transgenic mice groups a progressive severe non-purulent meningoencephalitis with astrogliosis and activation of microglia was noted; only astrocytes were activated in the wild-type mice (Figs. 9B–I, 10, 11). Interestingly, homozygous TNF-transgenic animals showed higher inflammatory scores than heterozygous BDV-infected mice at all time points investigated. Heterozygous and homozygous TNF-transgenic BDV-infected mice showed a significant increase of invading immune cells consisting of macrophages, T-cells and B-cells (data not shown) until 49 dpi (Tg/– p = 0.004; Tg/Tg p = 0.0024). Statistically significant differences in the inflammatory reaction between BDV-infected transgenic animals and BDV-infected non-transgenic mice were detected from day 35 p.i. on (35 dpi, –/– vs. Tg/Tg: p = 0.0357; 42 dpi –/– vs. Tg/–: p = 0.0256, –/– vs. Tg/Tg: p = 0.0416; 49 dpi –/– vs. Tg/–: p = 0.0281, –/– vs. Tg/Tg: p = 0.0416, table S2). Infiltration with mononuclear cells was present in all groups from 21 dpi on and was mainly found perivascularly in all three BDV-infected animal groups with some additional parenchymal invasion only in the transgenic animals starting 28 dpi. Immune cell infiltrates were predominantly located in all cortical regions investigated, striatum and thalamus. Both TNF-transgenic mice groups showed surprisingly little inflammatory reaction in the hippocampus. Even at the peak of the inflammatory response, the hippocampus displayed no or only mild immune cell infiltration in both TNF-transgenic groups despite high TNFtg and TNFto mRNA values (see above). Despite the remarkable inflammatory lesions in transgenic BDV-infected mice, neither in TNF-transgenic nor in non-transgenic animals, obvious neuronal necrosis was present in any brain region investigated, even not in the hippocampus. This was furthermore confirmed by Kresyl-violet staining (data not shown).

In the HE-stained sections, a significant increase of **microglia** occurred only in homozygous (p = 0.0105) and heterozygous (p = 0.0007) BDV-infected TNF-transgenic mice starting 21 dpi. (Figs. 9D–F, 11, table S2) which was strongest in the homozygous mice. At 42 and 49 dpi, in both transgenic BDV-infected mice groups and additionally at 28 dpi in Tg/Tg animals, the numbers of activated microglia were significantly higher than in wild-type BDV-infected animals (28 dpi Tg/Tg: p = 0.0210; 42 dpi Tg/–: p = 0.0265, Tg/Tg: p = 0.0436; 49 dpi Tg/–: p = 0.0179, Tg/Tg: p = 0.0436, table S2). Non-transgenic BDV-infected animals showed only occasionally rod shaped activated microglia up to 49 dpi. Rod shaped cells regularly expressed mac-1α. At day 42 p.i., the immunostained perivascular microglia/macrophages represent 37.6% in the –/–, 34.7% in the Tg/– and 37.8% in the Tg/Tg mice of the total perivascular cell count.

From 14 days p.i. on, an **astrogliosis** was observed in both BDV-infected transgenic mice groups, but also in BDV-infected non-transgenic mice despite only mild inflammatory lesions (Figs. 9G–I, Fig. S1). However, between 21 and 42 dpi, the score for GFAP-expressing astrocytes was highest in the homozygous mice. In both transgenic mice groups, from 21 dpi on severely enlarged astrocytes with swollen nuclei were additionally found adjacent to the inflammatory infiltrates. Some of these cells displayed signs of apoptosis. Statistically, both transgenic animal groups displayed significantly more GFAP-positive astrocytes than non-transgenic BDV-infected mice (table S2) at 28 dpi (Tg/–: p = 0.0471, Tg/Tg: p = 0.0177) and 35 dpi (Tg/–: p = 0.0400, Tg/Tg: p = 0.0443).

**Figure 12:**
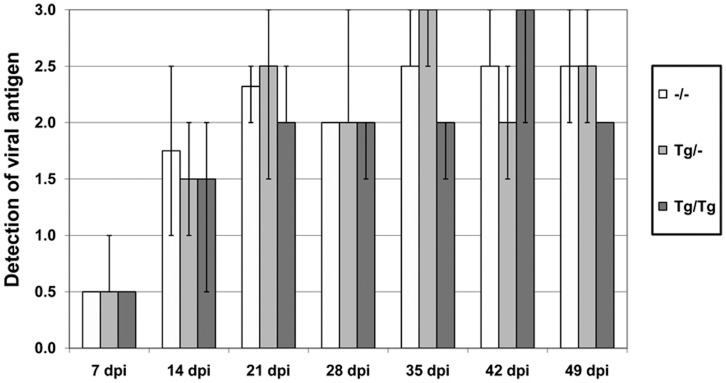
Detection of viral antigen in the brain of BDV-infected animals. After 21 dpi, the viral nucleoprotein BDV-N showed a disseminated and comparable distribution in the entire brain in all TNF-transgenic and wild-type animals. Score for the BDV-N immunoreactivity: 0: no detection of antigen; 1: single foci with BDV-N positive cells in some brain areas, <80 cells per HPF; 2: less than 150 positive cells per HPF, neuropil reaction, 3: more than 150 positive cells per HPF, distinct neuropil reaction p.i.: post infection, –/–: non-transgenic mice, Tg/–: heterozygous transgenic mice, Tg/Tg: homozygous transgenic mice, median, bar: minimal/maximal value.

The **viral nucleoprotein (BDV-N)** was detected 7 dpi in single neurons in the cerebral cortex and hippocampus in all BDV-infected mice groups, (Figs. 9J–L, 12). Between 21 and 49 dpi, BDV-N showed a disseminated distribution in the entire brain in all infected mice cohorts reaching the maximum of positive cells between 35 and 42 dpi. BDV-N was present in neurons, astrocytes, oligodendrocytes and single ependymal cells. In all cell types, the viral protein was found in the cytoplasm, nucleus and processes as well in as in the neuropil from 21 dpi on. Moreover, comparable quantity of BDV-N positive cells was present in TNF-overexpressing brain areas compared to brain regions with low or no transgenic TNF-expression in both transgenic groups.

**Infectious virus** was isolated on 28 and 49 days p.i. in TNF-transgenic and wild-type mice in similar titres ranging from 2×10^3^ up to 6×10^3^ REB-ID_50_ in each group.

**The Spearman Rank Correlation,** testing the direction and strength of the relationship between two variables, displayed significant correlations between the three different mice groups (non-infected controls, mock-infected and BDV-infected animals) and degree of encephalitis (p<0.0001, r_s = _0.96159). The severity of encephalitis showed a correlation with the occurrence of seizures (p = 0.005, rs = 0.78515).

## Discussion

Due to the versatile effects of TNF, combination of TNF-overexpression and experimental infection with a neurotropic virus such as BDV is perfectly suitable to analyze the effect of elevated TNF levels on the pathogenesis and outcome of neurotropic virus infections. This might particularly affect clinical signs, e.g. presensitation for epileptic seizures, host immune response, reaction pattern of brain cells and viral spread in the CNS. Intriguing actions of TNF in the CNS range from induction of antiviral immune responses, proconvulsive and anticonvulsive effects up to neurodegenerative and neuroprotective properties [1]–[6], [10], [20], [42], [43], [47], [52]–[54]. Thus, a biological effect on BDV-infection could be anticipated.

In the TNF-overexpressing mouse model, epileptic seizures after BDV-infection were associated with a severe inflammatory reaction and glial cell activation in the brain. Interestingly, spontaneous seizures have only occasionally been noted in other TNF-transgenic mice models [10], [49]. The mice used in the present study express murine TNF under the control of the promoter of the receptor-subunit NR2B of the NMDA glutamate receptor that guaranteed TNF-overexpression restricted to specific forebrain areas where BDV is regularly present [25], [34], [52], [55]. This was confirmed by quantification of TNFtg mRNA copy numbers in cortex cerebri, striatum and hippocampus where these levels were significantly higher than in the cerebellum. The absent or very low copy numbers in the cerebellum reflect the age-dependent NR2B expression [56]. NR2B mRNA levels were not influenced by transgene expression or BDV-infection. Albeit considerable more severe inflammatory brain lesions in the homozygous infected mice, the separate analysis of each brain area at 42 dpi did not reveal significant differences in the TNFtg mRNA levels between heterozygous and homozygous mice. However, in another own study using total brain sections at mid hippocampal-thalamic levels at 21 and 42 dpi significantly higher TNFtg mRNA levels in homozygous mice were measured when compared to the heterozygous cohort in a global comparison (data not shown). This indicates that in the homozygous mice TNFtg mRNA up-regulation is stronger early p.i. which can result in the more pronounced appearance of inflammatory infiltrates, glial activation and seizures in this cohort. Interestingly, TNFtg was also able to induce native TNF mRNA, total TNF mRNA levels consisted of approximately 50% native TNF mRNA. Therefore, native TNF can further contribute to the transgenic TNF effects. However, TNF mRNA levels did not increase significantly after BDV-infection in the transgenic mice, approximately 2-fold higher values were measured only in the hippocampus in the transgenic mice. This indicates that the definite and significant TNF-effects were most likely mediated by induction of additional factors as shown by the significant increase of IL-1, TNFR1 and TNFR2 mRNA indicating a higher availability of both TNF receptors. In experimental infection with Theiler virus, TNF, IL-6 and associated inflammatory changes mainly contribute to the occurrence of acute seizures [20], [21].

There is increasing evidence that human epilepsy of various etiologies is related to chronic inflammatory processes in the CNS as cause and/or consequence of seizures [1], [3], [4], [6], [7], [12]. Thus, the encephalitis in the TNF-transgenic mice most likely represents one important key factor for seizure induction. The occurrence of seizures is furthermore influenced by the degree and extent of inflammation and participating cell types [3], [5] which can be substantiated in the TNF-transgenic mice as the Spearman rank correlation revealed a significant effect of the severity of encephalitis on the occurrence of seizures. In the BDV-infected TNF-transgenic mice, the amount of invading immune cells correlated with the level of TNF-expression indicating that TNF presensitizes a primarily less sensitive mouse brain for immune cell invasion. The latter has been postulated as further trigger in the epileptogenic process but seem to exert also neuroprotective properties [1], [57], [58]. In general, the C57Bl/6 mouse line has been described as less susceptible to BDV-induced disease but this strain develops regularly an immune mediated disorder and clinical disease in combination with a presensitizing event such as IL-12-overexpression [24], [36], [59], comparable to the TNF-transgenic mice. TNF effects are achieved by increasing the permeability of the CNS blood brain barrier with induction of intercellular adhesion molecule-1 and vascular cell adhesion molecule-1 [3], [38]. These adhesion molecules can also be upregulated in endothelial cells by seizures itself with subsequent activation of perivascular glial cells [4], [57]. In experimentally BDV-infected mice and rats, an up-regulation of native TNF in the brain is regularly observed, even very early after infection [31]–[33] which underlines the essential role of TNF for the following inflammatory events also in our mouse model. However, elevated TNF-levels might also act as anticonvulsant because an astrocytic TNF-overexpression prevented seizures in mice via the TNFR2 [10], [42]. Most studies confirmed this protective effect via TNFR2 whereas TNF signaling via TNFR1 exert proconvulsive effects in most experimental settings [2], [6], [42], [43]. In the TNF-transgenic mice, up-regulation of both TNFR mRNAs was detected after BDV-infection so that anticonvulsant and proconvulsive TNF effects might have been operative. In this respect it should be mentioned that signaling via each TNFR does not represent an isolated process but that TNFR cross talk influences the outcome of TNF effects [47]. Further studies are warranted to elucidate these multifunctional aspects of TNF expression.

Beside immune cell invasion CNS inflammation regularly comprises activation of microglia followed by astrocytic responses as present in the BDV-infected rats and TNF-transgenic mice [25], [34], [55]. This can subsequently serve as further cytokine source. The data confirmed the expected correlation of TNF-expression, severe encephalitis and microglia activation but did apparently not change the percentage of perivascular microglia/macrophages in the total count of invading immune cells. It is widely accepted that TNF is an important mediator for microglia activation, e.g. reduced microglia activation was found in TNF-knock out mice [60]. In BDV-infection microglia activation seems to depend on persistent infection of neurons and activation of astrocytes [61]. Microglial TNF and IL-1 production play an important role in models mimicking CNS infections in children as seizure predisposition [44], [45]. The early and strong microglia activation in the neonatally BDV-infected TNF-transgenic mice was possibly comparably involved. In contrast, the astrogliosis rather reflects an unspecific response to the virus infection since also wild-type mice developed astrocyte activation. However, astrogliosis was more severe in the transgenic animals during increase of inflammation in the CNS and only TNF-transgenic animal showed an unsual astrocytic hypertrophy. In contrast, in BDV-infected, severely diseased MRL mice only a swelling of astrocytes but no increase of GFAP-reactivity has been described [24]. In C57Bl/6 mice, astrogliosis also occurred after infection with the neurotropic rabies virus [62] so that the genetic background might influence the astrocytic response. Astrocytes have been attributed as important source of cytokines, mainly IL-1ß, in epileptic brain tissue [2], [3], [6], [10]. IL-1 mRNA was also significantly upregulated after BDV-infection, predominantly in TNF-overexpressing brain areas. Thus, the unusual astrocytic hypertrophy in these regions might result from an altered astrocytic reaction pattern with cytokine up-regulation, thereby also triggering seizure induction.

The encephalitis in the TNF-transgenic mice has most likely also increased the risk for seizures by altering excitability of neurons or neuromodulatory responses as shown for other seizure models [4], [6], [13], [14], [15], [18], [63]. There is increasing evidence that cytokines such as TNF can interfere with the excitability of neurons by mediating synaptic scaling, e.g. exocytosis of excitatory AMPA receptors and endocytosis of inhibitory GABA A receptors and via long term effects on gene expression involved in synaptic reorganization, neurogenesis and cell death [2], [6], [13], [63]. TNF is furthermore involved in control of glutamatergic gliotransmission by interaction with extrasynaptic NMDA receptors [43], [64]. However, TNF-transgenic mice are protected against glutamate excitoxicity mainly by up-regulation of TNFR2 signaling as shown in vitro and in a retinal ischemia model and high TNF levels more likely employ TNFR2 signaling [2], [42], [47], [48], [52], [65]. Interestingly, TNFR2 signaling pathway can be further stimulated by coactivation of NMDA receptors and this can serve as inherent protection against glutamate-induced injuries [48]. Phenotypically, higher extrasynaptic NMDA receptor currents have been measured in acute hippocampal slice of young TNF-transgenic mice (data not shown). Thus, high TNF mRNA values in transgenic regions could have protected neurons even in case of BDV-infection since no notable neuronal necrosis was detected in any of these areas despite strong inflammatory lesions. No or only mild inflammation was found in the hippocampus where levels of TNF mRNAs were highest. It should be noted that cytokines may also affect the threshold to seizures independent of cell death [10]. Since neuronal morphology was well preserved, disturbances of the excitability of neurons and/or neuromodulatory systems as shown for the anticonvulsant dynorphin system have to be further detailed and to put into context to potential glial dysfunctions [8], [9], [14], [18], [66].

Beside seizures, TNF-transgenic animals also gained less weight than the wild-type mice after BDV-infection. This was a general feature in all BDV-infected mice compared to mock-infected animals. A reduced weight gain after BDV-infection has been noted in neonatal or adult rats using the BDV strain that caused the typical neurological type of BD [55], [67]. For neonatally rats, it was assumed that the lack of weight gain was due to the virus infection itself but in experimental LCMV-infection of mice, weight gain correlated with CD4^+^ T-cell activation rather than with TNF expression [68]. Both opportunities could be involved in our mouse model.

Strikingly, the viral spread within the brain and isolation of infectious virus showed no obvious differences between the wild-type and TNF-transgenic infected mice. The distribution of BDV-N was similar as described for adult infected rats [25], [34], [55] and indicates a persistent virus infection even in TNF-overexpressing animals. A disseminated viral spread was also found in mice deficient of several cytokines, chemokines or their receptors (IFNγ, Fas/FasL, CXCL10, CXCR3, inducible NO-synthase) [35], [36]. Thus, BDV seems to adapt easily to different immunological conditions to ensure its spread and persistence in the CNS.

Taken together, this study showed that high cerebral levels of TNF in combination with a neurotropic virus infection caused spontaneous epileptic seizures indicating a cause-and-effect-relationship. In the present model, high levels of TNF presensitize the mice brain to enhanced CNS inflammation with glial cell activation accompanied by cytokine and TNFR up-regulation. This demonstrates the impact of chronically elevated cytokine levels and the complex neuro-immune interactions for seizure induction which can be analyzed in appropriate animal models such as the experimental BDV-infection of TNF-transgenic mice.

## Materials and Methods

### 1. Mice and Experimental BDV-infection

C57Bl/6 homozygous (Tg/Tg, n = 25) and heterozygous (Tg/–, n = 34) TNF-transgenic and non-transgenic (–/–, n = 30) mice (total n = 89, [52]) were infected neonatally by intracerebral injection of a mouse-adapted BDV-strain (10% BDV-infected brain suspension 10^3^ ID_50_/ml), kindly provided by P. Staeheli, Department of Virology, University of Freiburg, Germany into the frontal cerebral cortex. Mock-infected (n = 42, 14 mice per group) and non-infected animals (n = 67, both transgenic groups n = 22, non-transgenic mice n = 23) of the three experimental groups served as controls. Male and female mice were used in equal amounts. The studies were approved by the Regierungspräsidium Hannover according to the guide for the care and use of animals in the State of Lower Saxony (Application No. Az. 509.6-42502-03/679). This also implies that all efforts have been made to minimize suffering of any animal.

Infection rate was 100%. The mice were killed weekly between 7 and 49 days post infection (dpi) by using thiobarbiturate followed by cervical dislocation. The brains were removed immediately and cut sagitally, one half was fixed in formalin and embedded in paraffin, the other half was embedded in OCT™-compound (Sakura, Finetec Europe, Zoeterwoude, The Netherlands), shock frozen and stored at −80°C.

### 2. Clinical Examination

Clinical and neurological examinations were carried out weekly starting at 21 dpi. The investigations were performed according to routine examination scheme for small animals [69] and based on the results of the study done by Hallensleben et al. [24]. The body weight was measured and different tests for the presence of neurological deficits were performed: Activity, the gait on the grid and on a flat surface as well as the ability to balance on a stick were evaluated using semi quantitative scores by two independent investigators and are detailed in table 1.

**Table 1:** Scoring of the clinical-neurological examination.

Score	activity	gait on grid/flat surface	ability to balance on a stick	sensitivity	grasp/withdrawal reflex
**4**	/	/	severe disturbances of equilibrium, balancingwas not possible	/	/
**3**	severe hyperactivity	severe unsteady gait	severe unsteadiness	/	/
**2**	moderate hyperactivity	moderate unsteady gait	moderate unsteadiness	severe hyperesthesia	severe hyperreflexia
**1**	mild hyperactivity	mild unsteady gait	mild unsteadiness	mild hyperesthesia	mild hyperreflexia
**0**	**normal**	**normal**	**no problems**	**normal**	**normal**
**−1**	mild hypoactivity	/	/	mild hypoesthesia	mild hyporeflexia
**−2**	moderate hypoactivity	/	/	severe hypoesthesia	severe hyporeflexia
**−3**	apathy	/	/	/	/

### 3. DNA Isolation and Quantitative PCR (qPCR)

For the analysis of the transgenic status of the mice, DNA was isolated from mouse tails using the E.N.Z.A.® Tissue DNA Mini Kit (Peqlab, Erlangen, Germany) according to the manufacturer’s protocol. The transgenic status of the animals was determined using the Comparative Quantitation Protocol of the Mx 4000 Multiplex Quantitative PCR System (Stratagene®, La Jolla, CA) and SYBR-Green® (Stratagene®) as fluorophore. DNA specimens of a known homozygous animal served as calibrator, glyceraldehyde-3-phosphate dehydrogenase (GAPDH) levels were used as an internal standard. One defined animal of each transgene status (homozygous, heterozygous and non-transgenic) was used in each run as control beside no template controls. All samples and controls were applied as duplicates in each run. The analysis was performed with primers detecting specifically the transgenic TNF (251 bp, forward: CTG GAT ATT CCC AAC ATG CG, reverse: CCC CGA ACG TCA GTA GAC AG; [50]) and primers specific for the murine GAPDH (288 bp, **GI: 6679936**, forward: GAG GCC GGT GCT GAG TAT GT, reverse: GGT GGC AGT GAT GGC ATG GA) using 40 cycles with 55°C as annealing temperature. A relative quantity (dRN  = 2^−ΔΔCt^) of 0 characterized non-transgenic mice. Heterozygous animals had a dRN of 0.3–0.7, whereas homozygous animals showed a dRN ≥0.8.

### 4. RNA Isolation and Quantitative RT-PCR

Highly transgenic TNF expressing brain areas such as cortex cerebri, hippocampal formation and striatum were excised from a sagittally cut brain section (42 dpi, n = 3 per group, approximately bregma −2.18 mm, interaural 1.62 mm [70]) of BDV-infected and non-infected mice. The cerebellum served as control brain region. New sterile scalpels were used for every brain region and mouse to avoid any contamination. RNA was isolated from these brain regions using Trizol® Reagent (Invitrogen, Karlsruhe, Germany) and purified using the RNeasy Minikit (Qiagen, Hilden, Germany) following the protocols of the supplier. After a digestion step with DNase I (Qiagen, Hilden, Germany), RNA was resuspended in 30 µl of RNAse-free water. Reverse transcription was performed applying the Omniskript® RT Kit (Qiagen, Hilden, Germany) and random primers (Promega, Madison) as described elsewhere [71]. The reaction took place in a final volume of 19 µl in buffer containing 0.2 mM dNTP mix, 0.5 µl Random Primer primer (Promega, Madison), 10 units RNaseOut™ Recombinant Ribonuclease Inhibitor (Invitrogen™ GmbH, Karlsruhe, Germany), 4 units of Omniskript® enzyme and 12 µl of total RNA.

Real-time quantitative RT-PCR analysis for the mRNA of transgenic TNF (TNFtg) applying the primer pairs used for the analysis of the transgene status (see before), total TNF (TNFto, 203 bp, **NM013693**, forward: GCC TCT TCT CAT TCC TGC TT, reverse: CAC TTG GTG GTT TGC TAC GA), IL-1α (179 bp, **NM010554,** forward: AAG CAA CGG GAA GAT TCT GA, reverse: TGA CAA ACT TCT GCC TGA CG), TNFR1 (197 bp, **NM011609,** forward: CAG TCT GCA GGG AGT GTG AA, reverse: CAC GCA CTG GAA GTG TGT CT), TNFR2 (171 bp, **NM011610**, forward: TAC CAA GGG TGG CAT CTC TC, reverse: TCC TGG GAT TTC TCA TCA GG), NR2B (199 bp, **NM008171** forward: TCC GAA GCT GGT GAT AAT CC, reverse: GAG AGG GTC CAC ACT TTC CA) and for the housekeeping genes glyceraldehyde-3-phosphate dehydrogenase (GAPDH, 288 bp, **BC085275**, forward: GAG GCC GGT GCT GAG TAT GT, reverse: GGT GGC AGT GAT GGC ATG GA), hypoxanthine phosphoribosyl-transferase-1 (HPRT, 169 bp, **BC083145**, forward: GGA CCT CTC GAA GTG TTG GA, reverse: TTG CGC TCA TCT TAG GCT TT) and β-actin (233 bp, **NM007393**, forward: GGC TAC AGC TTC ACC ACC AC, reverse: ATG CCA CAG GAT TCC ATA CC) gene were performed using the Mx3005® Multiplex Quantitative PCR System (Stratagene®) and SYBR-Green® (Stratagene®) as fluorophore as previously described [53]. The thermal profile was set as follows: 10 minutes at 95°C followed by 40 cycles with an annealing temperature of 60°C (TNFtg, TNFto, IL-1, TNFR1, TNFR2, NR2B, β-actin), 57°C (GAPDH) or 55°C (HPRT) for 60 seconds and 30 seconds at 72°C. Amplification of the cDNA was carried out using the Brilliant® QPCR Core Reagent Kit (Stratagene®). Estimation of the cDNA copy numbers for each gene was performed using a 10-fold serial dilution of appropriate cloned cDNA samples ranging from 10^7^ −10^1^ copies to generate standard curves as recently described [71]. The reference genes (GAPDH, HPRT and β-actin) were used for normalization of TNFtg, TNFto, TNFR1, TNFR2, IL-1 and NR2B mRNA copy numbers using geNorm (http://medgen.ugent.be/~jvdesomp/genorm/) according to Vandesompele et al. [72].

### 5. Histology

Formalin-fixed and paraffin-embedded brain sections were stained with hematoxylin and eosin (H&E) to assess the degree of encephalitis as defined by the presence of histopathological changes and mononuclear immune cell infiltrates at all time points investigated. The distribution of the lesions was investigated employing at least nine different brain regions including regions with high TNF-overexpression (cortex cerebri, striatum, hippocampus, thalamus), and low TNF-overexpression (hypothalamus, amygdala, mesencephalon) and non-expressing areas (cerebellum, medulla oblongata). The inflammatory reaction of leptomeningeal, perivascular and parenchymal infiltrates was scored within a 400-fold magnification as follows: 0: no inflammatory lesions; 1 (mild inflammation): up to 4 perivascular or leptomeningeal immune cells, perivascular cuffs with up to one layer of invading immune cells and up to 5 activated microglia surrounding the respective infiltrate; 2 (moderate inflammation): seven and more perivascular or leptomeningeal immune cells, perivascular cuffs of mostly 1–2 layers of invading immune cells and up to 20 activated microglia surrounding the particular infiltrate; 3 (severe inflammation): many perivascular or leptomeningeal immune cells, additional parenchymal immune cell foci, perivascular cuffs with three and more layers of invading immune cells and more than 20 activated microglia surrounding the respective infiltrate. The number of reactive microglia cells adjacent to the perivascular immune cell infiltrates in H&E-stained sections (cells with rod-shaped nuclei, perineuronal location) was counted in one high power field (HPF, 400-fold magnification) focusing the respective vessel in the center of the HPF. The average number per animal was calculated. The microglia origin of the rod shaped cells was confirmed by immunostaining with an antibody against mac-1α (eBioscience, San Diego, CA, 1∶3000 in PBS with 1% bovine serum albumine [BSA]; see below).

### 6. Immunohistochemistry (IHC)

Macrophages and microglia were recognized using a primary rat monoclonal antibody (mac-1α; eBioscience, San Diego, 1∶3000 in PBS with 1% BSA). The IHC was performed using serial frozen tissue sections and the ABC method (ABC Elite Standard [PK-6100], Vector Laboratories, Burlingame, CA) with 3,3′-diamonobenzidine (DAB) as substrate, adapting the protocols for paraffin embedded slides [34] and making the required adjustments. The sections were fixed for 10 minutes in acetone and processed without the unnecessary steps of deparaffination and antigen unmasking. The primary antibody was incubated for 1 h at room temperature. For quantitative evaluation of the percentage of perivascular inflammatory cells, immunostained cells were determined 42 dpi. The total numbers of cells in the cuffs were used as denominator and for each mouse brain. About 200 cells were counted followed by calculation of the particular percentage of mac-1α positive cells. The inflammatory infiltrates were counted in at least six different brain regions (cortex cerebri, striatum, hippocampus, thalamus, hypothalamus, amygdala) in all BDV-infected groups.

IHC using antibodies specific for glial fibrillary acidic protein (GFAP) and the BDV-nucleoprotein (BDV-N) was performed at all time points investigated using paraffin-embedded tissue sections and the ABC method (ABC Elite Standard [PK-6100], Vector Laboratories) with 3,3′-diamonobenzidine (DAB) as substrate as previously described [55]. GFAP was detected using a polyclonal rabbit antibody (DakoCytomation, Hamburg, Germany, 1∶1000 in PBS with 20% normal swine serum) and BDV-infection was confirmed using a monoclonal anti-BDV-N antibody (Bo18; 1∶500 in PBS with 1% BSA). The distribution and density of cells expressing GFAP and BDV-N was investigated employing at least nine different brain regions (cortex cerebri, striatum, hippocampus, thalamus, hypothalamus, amygdala, mesencephalon, cerebellum, medulla oblongata) in Tg/Tg, Tg/– and –/– animals of the non-infected, BDV-infected and mock-inoculated mice groups.

Semiquantitiative analysis of GFAP expression was performed using 100-fold and 400-fold magnification and was scored as follows: 0: no GFAP expressing cells; 1 (mild astrogliosis): less than 30 positive cells per HPF, positive cells mainly in cortex cerebri, hippocampus and striatum; 2 (moderate astrogliosis): 30–60 positive cells per HPF, some astrocytes exhibit swollen nuclei; 3 (severe astrogliosis): more than 60 positive cells per HPF. Semiquantitiative analysis of the BDV-N immunoreactivity was performed using 100-fold and 400-fold magnification and was evaluated as follows: 0: no detection of antigen; 1: single foci with BDV-N positive cells in some brain areas, less than 80 cells per HPF, no reaction within the neuropil; 2: less than 150 positive cells per HPF, neuropil reaction, BDV-N positive cells in nearly all brain regions; 3: more than 150 positive cells per HPF, distinct neuropil reaction, BDV-N positive cells in all brain regions investigated.

### 7. Detection of Infectious Virus

The isolation of infectious virus from fresh frozen TissueTek-embedded brain tissue of BDV-infected TNF-transgenic and wild-type mice (28 and 49 days p.i.) and determination of the virus titre were performed employing susceptible fetal rabbit brain cells; visualization was done by indirect immunofluorescence techniques (IIFT) according to previous protocols [73]. The virus titre was determined as REB-ID_50_ (infectious dose 50 for embryonic rabbit brain cells).

### 8. Statistics

The statistical evaluation of weight gain was performed using the analysis of variance for repeated measurements concerning the mean weight gain of each mouse group and infection (table S1). Additionally, a three-way analyses including three two-way and three one-way analysis of variance were carried out for the evaluation of TNFtg, TNFto, IL-1, TNFR1, TNFR2 and NR2B mRNA levels correlating brain region, transgene expression and infection (tables S3, S4). To achieve normal distribution, these data were logarithmically transformed. Furthermore, for comparison of gene copy numbers within the mouse groups, Tukey’s post-hoc test for multiple pairwise comparisons was applied. The other parameters of the clinical examinations, the results of the histological scoring, the activation of microglia and astrocytes as well as the results of the immunohistological staining for BDV-N and GFAP of the different mice groups as well as comparison of the different time points of infection within one group were analyzed by using Wilcoxon Scores and Kruskal-Wallis Tests (table S2). The Spearman rank correlation coefficient was used to calculate for relationship of the status of infection, occurrence of seizures and degree of inflammation. For all statistical analysis, differences were considered significant if *P-value*s were <0.05. All statistical calculations were performed using the statistical software SAS® 9.1 (SAS Institute Inc., Cary, NC).

## Supporting Information

Figure S1 **GFAP-immunostaining of hippocampi of BDV-infected animals.** Astrogliosis occured in all BDV-infected mice groups but was more servere in transgenic animals. There were no differences between hippocampal areas such as CA1 and dentate gyrus. 42 dpi, bar: 50 µm, –/–: non-transgenic mice, Tg/–: heterozygous transgenic mice, Tg/Tg: homozygous transgenic mice.
(PDF)

Table S1 **Statistics of weight gain.** The statistical evaluation of weight gain was performed using the analysis of variance for repeated measurements concerning the mean weight gain of each mouse group and infection. The respective p-values are given. –/–: non-transgenic mice, Tg/–: heterozygous transgenic mice, Tg/Tg: homozygous transgenic mice.
(PDF)

Tables S2 **Statistics of clinical investigation, encephalitis and glia activation.** The clinical examinations, the histological scoring and the activation of microglia and astrocytes of the different mice groups as well as comparison of the different time points of infection within one group were analyzed by using Wilcoxon Scores and Kruskal-Wallis Tests. The respective p-values are given. –/–: non-transgenic mice, Tg/–: heterozygous transgenic mice, Tg/Tg: homozygous transgenic mice.
(PDF)

Table S3 **Statistics of qRT-PCR assays.** A three-way analyses including three two-way and three one-way analysis of variance were carried out for the evaluation of TNFtg, TNFto, IL-1, TNFR1, TNFR2 and NR2B mRNA levels correlating brain region, transgene expression and infection. To achieve normal distribution, these data were logarithmically transformed. For comparison of gene copy numbers within the mouse groups, Tukey’s post-hoc test for multiple pairwise comparisons was applied. The respective p-values are given. –/–: non-transgenic mice, Tg/–: heterozygous transgenic mice, Tg/Tg: homozygous transgenic mice.
(PDF)

Table S4 **Additional statistical information of qRT-PCR assays.** A three-way analyses including three two-way and three one-way analysis of variance were carried out for the evaluation of TNFtg, TNFto, IL-1, TNFR1, TNFR2 and NR2B mRNA levels correlating brain region, transgene expression and infection. As additional information p-values were completed by F-values and degree of freedom. –/–: non-transgenic mice, Tg/–: heterozygous transgenic mice, Tg/Tg: homozygous transgenic mice.
(PDF)
