# Supplementary figures and images for: TNF-Overexpression in Borna Disease Virus-Infected Mouse Brains Triggers Inflammatory Reaction and Epileptic Seizures
Source: PLoS One. 2012 Jul 25;7(7):e41476. doi: 10.1371/journal.pone.0041476 (PMC3405098; doi:10.1371/journal.pone.0041476)

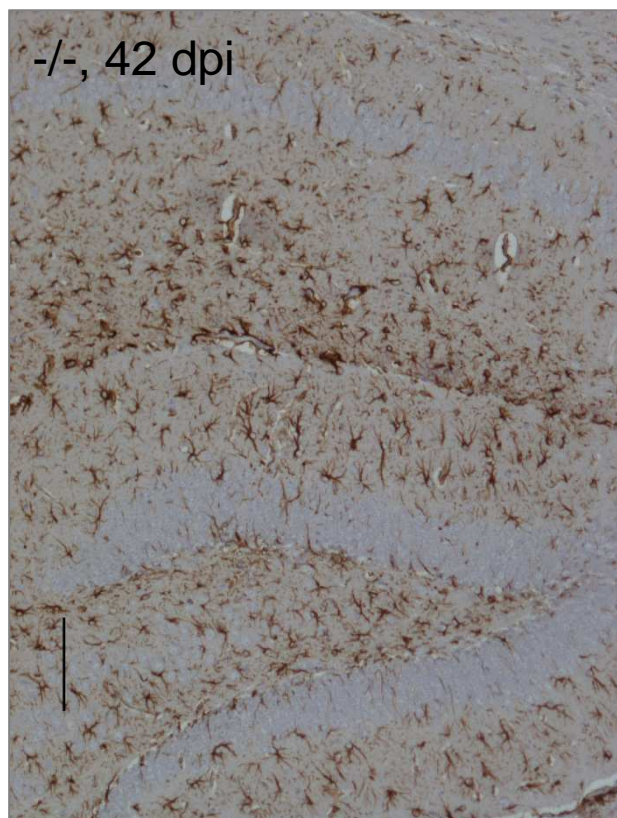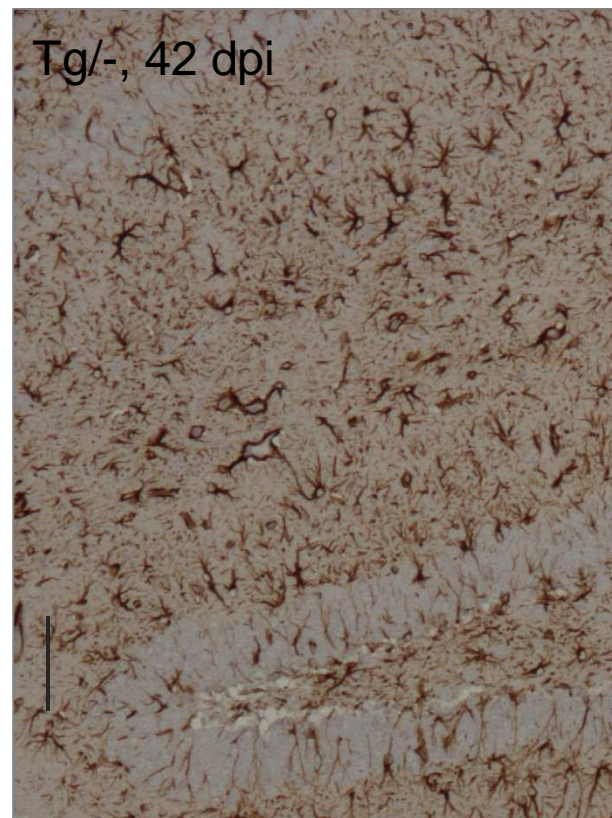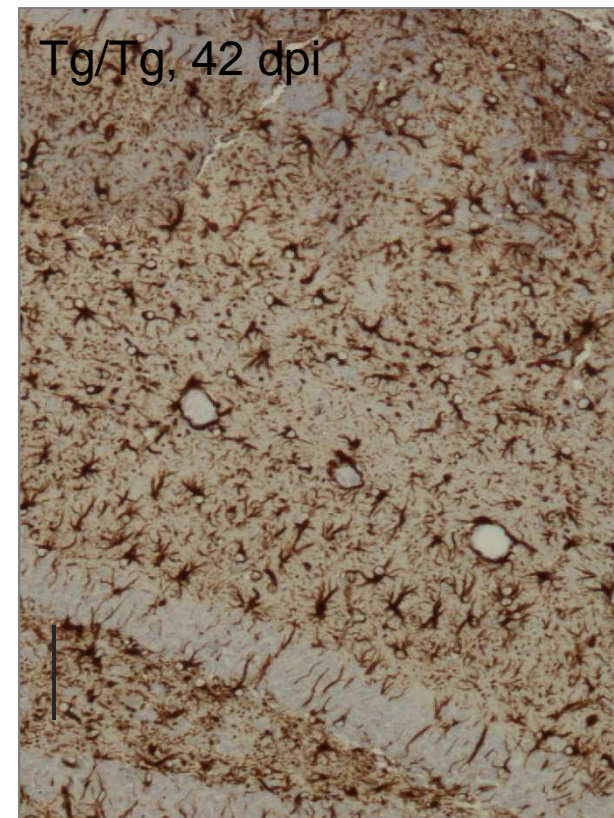

Supplement: Figure S1 — GFAP-immunostaining of hippocampi of BDV-infected animals. Astrogliosis occured in all BDV-infected mice groups but was more servere in transgenic animals. There were no differences between hippocampal areas such as CA1 and dentate gyrus. 42 dpi, bar: 50 µm, –/–: non-transgenic mice, Tg/–: heterozygous transgenic mice, Tg/Tg: homozygous transgenic mice. (PDF) [file pone.0041476.s001.pdf]
