# Supplementary material for: TNF-Overexpression in Borna Disease Virus-Infected Mouse Brains Triggers Inflammatory Reaction and Epileptic Seizures
Source: PLoS One. 2012 Jul 25;7(7):e41476. doi: 10.1371/journal.pone.0041476 (PMC3405098; doi:10.1371/journal.pone.0041476)
Supplement: Table S1 — Statistics of weight gain. The statistical evaluation of weight gain was performed using the analysis of variance for repeated measurements concerning the mean weight gain of each mouse group and infection. The respective p-values are given. –/–: non-transgenic mice, Tg/–: heterozygous transgenic mice, Tg/Tg: homozygous transgenic mice. (PDF) [file pone.0041476.s002.pdf]

status of infection	0.0091
transgenic status	0.0814
status of infection x transgenic status	0.7760
BDV -/- - mock -/-	0.0101
BDV Tg/- - mock Tg/-	0.8459
BDV Tg/Tg - mock Tg/Tg	0.0275
BDV -/- - BDV Tg/-	0.4925
BDV -/- - BDV Tg/Tg	0.0450
BDV Tg/- - BDV Tg/Tg	0.0128
mock -/- - mock Tg/-	0.0763
mock -/- - mock Tg/Tg	0.1657
mock Tg/- - mock Tg/Tg	0.6552
