# Supplementary material for: TNF-Overexpression in Borna Disease Virus-Infected Mouse Brains Triggers Inflammatory Reaction and Epileptic Seizures
Source: PLoS One. 2012 Jul 25;7(7):e41476. doi: 10.1371/journal.pone.0041476 (PMC3405098; doi:10.1371/journal.pone.0041476)
Supplement: Tables S2 — Statistics of clinical investigation, encephalitis and glia activation. The clinical examinations, the histological scoring and the activation of microglia and astrocytes of the different mice groups as well as comparison of the different time points of infection within one group were analyzed by using Wilcoxon Scores and Kruskal-Wallis Tests. The respective p-values are given. –/–: non-transgenic mice, Tg/–: heterozygous transgenic mice, Tg/Tg: homozygous transgenic mice. (PDF) [file pone.0041476.s003.pdf]

microglia activation - global time course comparision of the BDV infected mouse groups

Kruskal-Wallis test

-/-	Tg/-	Tg/Tg
0.0680	0.0007	0.0105

microglia activation - comparision between the BDV infected mouse groups

dpi	Kruskal-Wallis test	Wilcoxon test -/- - Tg/-	-/- - Tg/Tg	Tg/- - Tg/Tg
21	0.0560	-	-	-
28	0.0297	0.0716	0.0210	0.2395
35	0.0799	-	-	-
42	0.0184	0.0265	0.0436	0.2159
49	0.0155	0.0179	0.0436	0.2330

astrogliosis / GFAP staining - comparision between the BDV infected mouse groups

dpi	Kruskal-Wallis test	Wilcoxon test -/- - Tg/-	-/- - Tg/Tg	Tg/- - Tg/Tg
21	0.1127	-	-	-
28	0.0139	0.0471	0.0177	0.1814
35	0.0184	0.0400	0.0443	0.1175
42	0.1950	-	-	-
49	0.1009	-	-	-

Encephalitis score - comparision between the BDV infected mouse groups

dpi	Kruskal-Wallis test	Wilcoxon test<br>-/- - Tg/-	-/- - Tg/Tg	Tg/- - Tg/Tg
21	0.1216	-	-	-
28	0.1047	-	-	-
35	0.0563	-	-	-
42	0.0130	0.0256	0.0416	0.0955
49	0.0124	0.0281	0.0416	0.0606

Clinical investigation comparision of the status of infection groups after BDV infection

dpi	activity<br>Kruskal-Wallis test	gait on the grid<br>Kruskal-Wallis test	gait on a flat surface<br>Kruskal-Wallis test	Wilcoxon test<br>-/- - Tg/-	-/- - Tg/Tg	Tg/- - Tg/Tg	balance on a stick<br>Kruskal-Wallis test
21	0.5790	0.6086	1.000	-	-	-	0.1573
28	0.9703	0.7071	0.7558	-	-	-	0.3184
35	0.1816	0.7297	0.5238	-	-	-	0.9555
42	0.1108	0.4391	0.0389	0.4237	0.1056	0.0416	0.9281
49	0.7280	0.7877	0.4236	-	-	-	0.3501

Clinical investigation comparision between non transgenic mice after BDV infection and controls

Wilcoxon test

dpi	activity	gait on the grid	gait on a flat surface	balance on a stick
21	1.000	0.1336	1.000	1.000
28	1.000	0.8308	0.7518	0.7237
35	0.1949	0.3340	0.7518	0.1514
42	0.3017	0.1719	0.6056	0.1175
49	0.7518	0.1595	0.4687	0.1595

Clinical investigation comparision between heterozygous transgenic mice after BDV infection and controls

Wilcoxon test

dpi	activity	gait on the grid	gait on a flat surface	balance on a stick
21	0.1904	0.0759	0.1489	1.000
28	0.8257	0.1555	0.4624	0.1876
35	0.0641	1.000	1.000	0.0416
42	0.4017	0.2801	1.000	0.4113
49	0.8611	0.0715	0.5445	0.2108

Clinical investigation comparision between homozygous transgenic mice after BDV infection and controls

Wilcoxon test

dpi	activity	gait on the grid	gait on a flat surface	balance on a stick
21	0.6926	0.1336	1.000	1.000
28	0.8057	0.0732	0.4624	0.5338
35	0.3894	0.5224	0.6056	0.2207
42	0.4142	0.4677	0.2188	0.1281
49	1.000	0.2128	0.3329	0.6386
