# Supplementary material for: TNF-Overexpression in Borna Disease Virus-Infected Mouse Brains Triggers Inflammatory Reaction and Epileptic Seizures
Source: PLoS One. 2012 Jul 25;7(7):e41476. doi: 10.1371/journal.pone.0041476 (PMC3405098; doi:10.1371/journal.pone.0041476)
Supplement: Table S3 — Statistics of qRT-PCR assays. A three-way analyses including three two-way and three one-way analysis of variance were carried out for the evaluation of TNFtg, TNFto, IL-1, TNFR1, TNFR2 and NR2B mRNA levels correlating brain region, transgene expression and infection. To achieve normal distribution, these data were logarithmically transformed. For comparison of gene copy numbers within the mouse groups, Tukey’s post-hoc test for multiple pairwise comparisons was applied. The respective p-values are given. –/–: non-transgenic mice, Tg/–: heterozygous transgenic mice, Tg/Tg: homozygous transgenic mice. (PDF) [file pone.0041476.s004.pdf]

		TNFtg	TNFto	IL-1	TNFR1	TNFR2	NR2B-receptor
**multiple analysis of variance including dependent (brain region) and independent (transgenic status, status of infection) variables**	Brain region	<.0001	<.0001	<.0001	0.0069	<.0001	<.0001
	Transgenic status	0.5051	<.0001	0.0002	0.1103	0.0031	0.2386
	Status of infection	0.6046	<.0001	<.0001	<.0001	<.0001	0.9084
	Brain region x transgenic status	0.7269	<.0001	0.0061	0.4739	0.0018	0.8227
	Brain region x status of infection	0.4459	0.0873	0.0019	0.218	0.442	0.4661
	status of infection x transgenic status	0.5595	<.00001	0.341	0.874	0.1595	0.3751
	Brain region x transgenic status x status of infection	0.93	0.2837	0.4653	0.1215	0.2507	0.6697
hoc-test	Hippocampus -/- BDV - Hippocampus Tg/Tg BDV	-	<.0001	0.0047	0.2807	0.0287	0.4445
	Hippocampus -/- BDV - Hippocampus Tg/- BDV	-	<.0001	0.005	0.0594	0.0082	0.1554
	Hippocampus -/- BDV - Hippocampus -/- mock	-	<.0001	0.0033	0.0391	0.0515	0.4392
	Hippocampus -/- BDV - Cortex -/- BDV	-	0.0394	0.7472	0.3296	0.9416	0.0457
	Hippocampus -/- BDV - Cerebellum -/- BDV	-	0.213	0.7047	0.4162	0.2839	<.0001
	Hippocampus -/- BDV - Striatum -/- BDV	-	0.0123	0.0904	0.9487	0.5117	0.2419
	Hippocampus Tg/Tg BDV - Hippocampus Tg/- BDV	0.9769	0.5169	0.4399	0.4	0.6073	0.5021
	Hippocampus Tg/Tg BDV - Hippocampus Tg/Tg mock	0.357	0.1488	0.0347	0.0019	0.0268	0.7378
	Hippocampus Tg/Tg BDV - Cortex Tg/Tg BDV	0.0694	0.1442	0.8476	0.9176	0.8118	0.4586
	Hippocampus Tg/Tg BDV - Cerebellum Tg/Tg BDV	0.0018	<.0001	0.0028	0.0745	0.0013	<.0001
	Hippocampus Tg/Tg BDV - Striatum Tg/Tg BDV	0.5761	0.2615	0.2194	0.1589	0.0502	0.1549
	Hippocampus Tg/- BDV - Hippocampus Tg/- mock	0.5218	0.3018	0.0002	<.0001	0.0026	0.0627
	Hippocampus Tg/- BDV - Cortex Tg/- BDV	0.0552	0.1161	0.193	0.0741	0.3376	0.6433
	Hippocampus Tg/- BDV - Cerebellum Tg/- BDV	0.0007	<.0001	0.0004	0.0364	0.0033	<.0001
	Hippocampus Tg/- BDV - Striatum Tg/- BDV	0.7263	0.3817	0.4104	0.5778	0.0161	0.0325
	Hippocampus -/- mock - Hippocampus Tg/Tg mock	-	<.0001	0.0003	0.9131	0.0549	0.7311
	Hippocampus -/- mock - Hippocampus Tg/- mock	-	<.0001	0.0068	0.6944	0.1237	0.2187
	Hippocampus -/- mock - Cortex -/- mock	-	0.8102	0.7204	0.7108	0.7905	0.1824
	Hippocampus -/- mock - Cerebellum -/- mock	-	0.9778	0.0008	0.5164	0.1343	<.0001
	Hippocampus -/- mock - Striatum -/- mock	-	0.983	0.1955	0.3118	0.6376	0.7816
	Hippocampus Tg/Tg mock - Hippocampus Tg/- mock	0.7973	0.822	0.281	0.7764	0.6859	0.3712
	Hippocampus Tg/Tg mock - Cortex Tg/Tg mock	0.8306	0.7771	0.9633	0.6311	0.9531	0.1008
	Hippocampus Tg/Tg mock - Cerebellum Tg/Tg mock	0.0098	<.0001	<.0001	0.5355	0.0026	<.0001
	Hippocampus Tg/Tg mock - Striatum Tg/Tg mock	0.3753	0.7052	0.2455	0.0604	0.5965	0.6867
	Hippocampus Tg/- mock - Cortex Tg/- mock	0.9772	0.8496	0.3342	0.8797	0.5984	0.1058
	Hippocampus Tg/- mock - Cerebellum Tg/- mock	0.0056	<.0001	<.0001	0.0663	0.0014	<.0001

pairwise comparison applying tuckey postr

Hippocampus Tg/- mock - Striatum Tg/- mock	0.7248	0.7817	0.824	0.0623	0.8627	0.4818
Cortex -/- BDV - Cortex Tg/Tg BDV	-	<.0001	0.006	0.0597	0.0396	0.6523
Cortex -/- BDV - Cortex Tg/- BDV	-	<.0001	<.0001	0.2662	0.0007	0.9694
Cortex -/- BDV - Cortex -/- mock	-	<.0001	0.0035	0.1297	0.0357	0.9035
Cortex -/- BDV - Cerebellum -/- BDV	-	0.0025	0.9142	0.0853	0.2674	<.0001
Cortex -/- BDV - Striatum -/- BDV	-	0.6434	0.2563	0.3172	0.5139	0.4145
Cortex Tg/Tg BDV - Cortex Tg/- BDV	0.4633	0.4501	0.1234	0.4205	0.1253	0.68
Cortex Tg/Tg BDV - Cortex Tg/Tg mock	0.9875	0.4501	0.0236	0.0086	0.0381	0.598
Cortex Tg/Tg BDV - Cerebellum Tg/Tg BDV	0.0294	<.0001	0.0036	0.0919	0.0028	<.0001
Cortex Tg/Tg BDV - Striatum Tg/Tg BDV	0.1024	0.7429	0.395	0.1459	0.0499	0.046
Cortex Tg/- BDV - Cortex Tg/- mock	0.2239	0.5337	0.0001	0.0069	<.0001	0.4217
Cortex Tg/- BDV - Cerebellum Tg/- BDV	0.062	<.0001	<.0001	0.6917	0.0004	<.0001
Cortex Tg/- BDV - Striatum Tg/- BDV	0.1325	0.5007	0.6897	0.2263	0.1727	0.0161
Cortex -/- mock - Cortex Tg/Tg mock	-	<.0001	0.0008	0.4797	0.0371	0.9647
Cortex -/- mock - Cortex Tg/- mock	-	<.0001	0.0021	0.8555	0.1863	0.3368
Cortex -/- mock - Cerebellum -/- mock	-	0.8444	0.0008	0.77	0.2153	<.0001
Cortex -/- mock - Striatum -/- mock	-	0.8371	0.4399	0.186	0.5104	0.3175
Cortex Tg/Tg mock - Cortex Tg/- mock	0.6303	0.8298	0.7108	0.3753	0.4192	0.3591
Cortex Tg/Tg mock - Cerebellum Tg/Tg mock	0.0164	0.0002	<.0001	0.8749	0.0036	<.0001
Cortex Tg/Tg mock - Striatum Tg/Tg mock	0.3042	0.5353	0.0037	0.1697	0.5985	0.2384
Cortex Tg/- mock - Cerebellum Tg/- mock	0.0056	<.0001	<.0001	0.0493	0.0062	<.0001
Cortex Tg/- mock - Striatum Tg/- mock	0.7211	0.6615	0.3335	0.0531	0.5316	0.3795
Cerebellum -/- BDV - Cerebellum Tg/Tg BDV	-	0.5212	0.9347	0.1285	0.0372	0.2983
Cerebellum -/- BDV - Cerebellum Tg/- BDV	-	0.9401	0.8956	0.3055	0.1995	0.33
Cerebellum -/- BDV - Cerebellum -/- mock	-	<.0001	<.0001	0.0009	<.0001	0.8528
Cerebellum -/- BDV - Striatum -/- BDV	-	0.0003	0.2118	0.4352	0.5841	<.0001
Cerebellum Tg/Tg BDV - Cerebellum Tg/- BDV	0.7025	0.5708	0.8313	0.6087	0.3972	0.9463
Cerebellum Tg/Tg BDV - Cerebellum Tg/Tg mock	0.8067	0.8026	<.0001	0.3787	0.0468	0.5823
Cerebellum Tg/Tg BDV - Striatum Tg/Tg BDV	0.0002	<.0001	<.0001	0.0019	<.0001	<.0001
Cerebellum Tg/- BDV - Cerebellum Tg/- mock	0.8719	0.9461	<.0001	0.6701	0.0012	0.6798
Cerebellum Tg/- BDV - Striatum Tg/- BDV	0.001	<.0001	<.0001	0.0989	<.0001	<.0001
Cerebellum -/- mock - Cerebellum Tg/Tg mock	-	<.0001	0.2619	0.2508	0.7443	0.7545
Cerebellum -/- mock - Cerebellum Tg/- mock	-	<.0001	0.1156	0.0387	0.7784	0.2316
Cerebellum -/- mock - Striatum -/- mock	-	0.9934	<.0001	0.0966	0.0354	<.0001
Cerebellum Tg/Tg mock - Cerebellum Tg/- mock	0.9812	0.3783	0.6394	0.3346	0.5443	0.3732
Cerebellum Tg/Tg mock - Striatum Tg/Tg mock	0.0007	<.0001	<.0001	0.2054	0.0002	<.0001

	Cerebellum Tg/- mock - Striatum Tg/- mock	0.0081	<.0001	<.0001	0.9667	0.0003	<.0001
	Striatum -/- BDV - Striatum Tg/Tg BDV	-	<.0001	0.0113	0.0209	0.0014	0.1009
	Striatum -/- BDV - Striatum Tg/- BDV	-	<.0001	0.0032	0.1869	<.0001	0.0936
	Striatum -/- BDV - Striatum -/- mock	-	<.0001	0.0015	0.2312	0.0362	0.9491
	Striatum Tg/Tg BDV - Striatum Tg/- BDV	0.385	0.6792	0.6287	0.292	0.3477	0.9694
	Striatum Tg/Tg BDV - Striatum Tg/Tg mock	0.5369	0.9533	0.0311	0.0069	0.0009	0.1726
	Striatum Tg/- BDV - Striatum Tg/- mock	0.5207	0.6318	<.0001	0.0431	<.0001	0.4247
	Striatum -/- mock - Striatum Tg/Tg mock	-	<.0001	0.0004	0.448	0.0493	0.8209
	Striatum -/- mock - Striatum Tg/- mock	-	<.0001	0.0823	0.6438	0.1968	0.4005
	Striatum Tg/Tg mock - Striatum Tg/- mock	0.3717	0.4057	0.0445	0.7652	0.4762	0.5374
