# Supplementary material for: TNF-Overexpression in Borna Disease Virus-Infected Mouse Brains Triggers Inflammatory Reaction and Epileptic Seizures
Source: PLoS One. 2012 Jul 25;7(7):e41476. doi: 10.1371/journal.pone.0041476 (PMC3405098; doi:10.1371/journal.pone.0041476)
Supplement: Table S4 — Additional statistical information of qRT-PCR assays. A three-way analyses including three two-way and three one-way analysis of variance were carried out for the evaluation of TNFtg, TNFto, IL-1, TNFR1, TNFR2 and NR2B mRNA levels correlating brain region, transgene expression and infection. As additional information p-values were completed by F-values and degree of freedom. –/–: non-transgenic mice, Tg/–: heterozygous transgenic mice, Tg/Tg: homozygous transgenic mice. (PDF) [file pone.0041476.s005.pdf]

	TNFtg			TNFto		
	p-value	F-value	Deg of free	p-value	F-value	Deg of free
Brain region	<.0001	21.68	24	<.0001	26.5	36
Transgenic status	0.5051	0.2900	8	<.0001	376.24	12
Status of infection	0.6046	0.4900	8	<.0001	50.97	12
Brain region x transgenic status	0.7269	0.4400	24	<.0001	14.32	36
Brain region x status of infection	0.4459	0.9200	24	0.0873	2.36	36
status of infection x transgenic status	0.5595	0.3700	8	<.00001	37.45	12
Brain region x transgenic status x status of infection	0.93	0.1500	24	0.2837	1.3	36
	IL-1			TNFR1		
	p-value	F-value	Deg of free	p-value	F-value	Deg of free
Brain region	<.0001	66.67	36	0.0069	4.73	36
Transgenic status	0.0002	17.9700	12	0.1103	2.6600	12
Status of infection	<.0001	88.0800	12	<.0001	59.3700	12
Brain region x transgenic status	0.0061	3.6600	36	0.4739	0.9500	36
Brain region x status of infection	0.0019	6.0700	36	0.218	1.5500	36
status of infection x transgenic status	0.341	1.1800	12	0.874	0.1400	12
Brain region x transgenic status x status of infection	0.4653	0.9600	36	0.1215	1.8300	36
	TNFR2			NR2B-receptor		
	p-value	F-value	Deg of free	p-value	F-value	Deg of free
Brain region	<.0001	27.1	36	<.0001	150.63	36
Transgenic status	0.0031	9.7000	12	0.2386	1.6200	12
Status of infection	<.0001	81.8900	12	0.9084	0.0100	12
Brain region x transgenic status	0.0018	4.4500	36	0.8227	0.4700	36
Brain region x status of infection	0.442	0.9200	36	0.4661	0.8700	36
status of infection x transgenic status	0.1595	2.1500	12	0.3751	1.0700	12
Brain region x transgenic status x status of infection	0.2507	1.3800	36	0.6697	0.6800	36

	weight gain		
	p-value	F-value	Deg of free
status of infection	0.0091	3.15	5
transgenic status	0.0814	1.69	10
status of infection x transgenic status	0.7760	0.64	10
